# VEGFR2-dependent VEGFR3 expression leads to CXCL1/2 and CXCR2 induction in the development of retinal neovascularization

**DOI:** 10.1007/s10456-026-10104-1

**Published:** 2026-10-25

**Authors:** Chandra Sekhar Yadavalli, Raj Kumar, Suresh Govatati, Gadiparthi N. Rao

**Affiliations:** Department of Physiology, University of Tennessee Health Science Center, 71 S Manassas Street, Memphis, TN 38163 USA

**Keywords:** CXCL1/2, CXCR2, IL-8, Neovascularization, Oxygen-induced retinopathy, VEGFR2/3

## Abstract

Elevated levels of IL-8 have been detected in the vitreous samples of patients with diabetic retinopathy. However, its role in retinal neovascularization remains unknown. We hypothesized that IL-8 and its murine orthologues, CXCL1/2, are key mediators of oxygen-induced retinopathy (OIR)-driven retinal angiogenesis. To this end, we found that VEGFA stimulates CXCL1/2 and their receptor CXCR2 expression in mouse retinal microvascular endothelial cells (MRMVECs) in a time-dependent manner. Recombinant CXCL1/2 induced MRMVEC migration, proliferation, sprouting, and tube formation alone and in combination. Neutralizing CXCL1/2 or CXCR2 antibodies (Abs) attenuated the angiogenic effects of CXCL1/2 as well as VEGFA. In line with these observations in mice, VEGFA induced IL-8 and its receptor CXCR2 expression in human retinal microvascular endothelial cells (HRMVECs), and neutralizing IL-8 or CXCR2 Abs suppressed their angiogenic effects. Furthermore, OIR induced CXCL1/2 and CXCR2 expression in the mouse retina in vivo, and blockade of their function using their neutralizing Abs reduced retinal neovascularization. Mechanistically, depletion of ATF6 or GRP78 levels abrogated VEGFA-induced CXCL1/2 and CXCR2 expression in MRMVECs and IL-8 and CXCR2 expression in HRMVECs in vitro and OIR-induced CXCL1/2 and CXCR2 expression in vivo in mice. Furthermore, silencing VEGFR2 or VEGFR3 levels inhibited VEGFA/OIR-induced ATF6-GRP78 activation and their downstream CXCL1/2, IL-8 and CXCR2 expression both in vitro and in vivo. Together, these findings identify IL-8 as potential therapeutic target for treating retinal neovascular diseases.

**Supplementary Information:**

The online version contains supplementary material available at https://doi.org/10.1007/s10456-026-10104-1.

## Introduction

Pathological retinal neovascularization is a major cause of vision impairment and blindness worldwide, contributing to ischemic diseases such as proliferative diabetic retinopathy (PDR), retinopathy of prematurity (ROP), and age-related macular degeneration (AMD) [1–3]. These conditions are characterized by abnormal growth of fragile and leaky blood vessels in the retina, leading to retinal detachment, hemorrhage, and fibrosis [4]. Despite advances in anti-angiogenic therapies, primarily those targeting vascular endothelial growth factor (VEGF), significant limitations remain, including incomplete response, need for repeated intravitreal injections, and potential adverse effects on normal vascular homeostasis [5, 6]. Thus, a deeper understanding of the molecular mechanisms underlying the pathological retinal neovascularization is critical for the development of more effective and targeted therapies.

Chemokines, a large family of small, secreted proteins that are crucial regulators of leukocyte trafficking [7], have recently been emerged as important modulators of angiogenesis [8, 9]. Among them, interleukin-8 (IL-8, aka CXCL8) has been well studied in the context of tumor angiogenesis, where it promotes endothelial cell migration, proliferation, and survival [10]. Elevated levels of IL-8 have been reported in the vitreous humor of patients with PDR, suggesting a potential role for it in retinal angiogenesis [11, 12]. However, the contribution of IL-8 to retinal neovascularization has remained largely unexplored. Mice, commonly used in experimental models of retinal neovascularization, do not express IL-8. Instead, they express its murine orthologues, CXCL1/2, and share functional similarities, including binding to the same receptor, namely, CXCR2 [13]. CXCL1/2 and IL-8 have been implicated in inflammatory processes and vascular pathologies in other tissues [14], but their role in the context of VEGF/OIR-driven retinal neovascularization is unknown.

Hypoxia is a potent driver of retinal neovascularization, largely through the upregulation of VEGF and activation of its receptors VEGFR2 and VEGFR3 [15, 16]. Apart from VEGF signaling, accumulating evidence suggests that cellular stress responses such as the unfolded protein response (UPR), are also activated under hypoxic conditions and can influence angiogenesis [17, 18]. Activating transcription factor 6 (ATF6) is a key sensor of the UPR pathway, which, upon activation, induces chaperones like glucose-regulated protein 78 (GRP78) to restore protein-folding homeostasis [19, 20]. Recently others and we have reported that UPR components, particularly ATF6 and GRP78 mediate pro-angiogenic signaling events in tumor angiogenesis and retinal neovascularization [21, 22]. We have also shown that VEGFR2-dependent VEGFR3 expression was required for VEGFA/OIR-induced ATF6-GRP78 signaling activation in the mediation of pathological retinal angiogenesis [22]. However, it is relatively unknown whether VEGFA induces cytokines CXCL1/2, if so, the link between ATF6-GRP8 signaling and these chemokines in the mediation of pathological angiogenesis. Therefore, in this study, we tested the hypothesis that VEGF/OIR-induced activation of the ATF6-GRP78 arm of the UPR leads to upregulation of CXCL1/2 in mice and IL-8 in humans, which then mediate key endothelial cell angiogenic responses. Using a combination of in vitro functional assays in mouse and human retinal microvascular endothelial cells (MRMVECs & HRMVECs, respectively) and an in vivo OIR model, we delineate the mechanistic signaling events linking VEGF-VEGFR, UPR components, chemokine induction, and retinal neovascularization. Our findings provide new insights into the complex interplay between VEGFR2/3, ATF6-GRP78 and chemokines in the modulation of pathological retinal angiogenesis and suggest that IL-8 may serve as novel therapeutic strategy for treating retinal neovascular diseases.

## Results

### CXCL1/2 mediate VEGFA-induced angiogenic events in mouse retinal microvascular endothelial cells

**Fig. 1:**
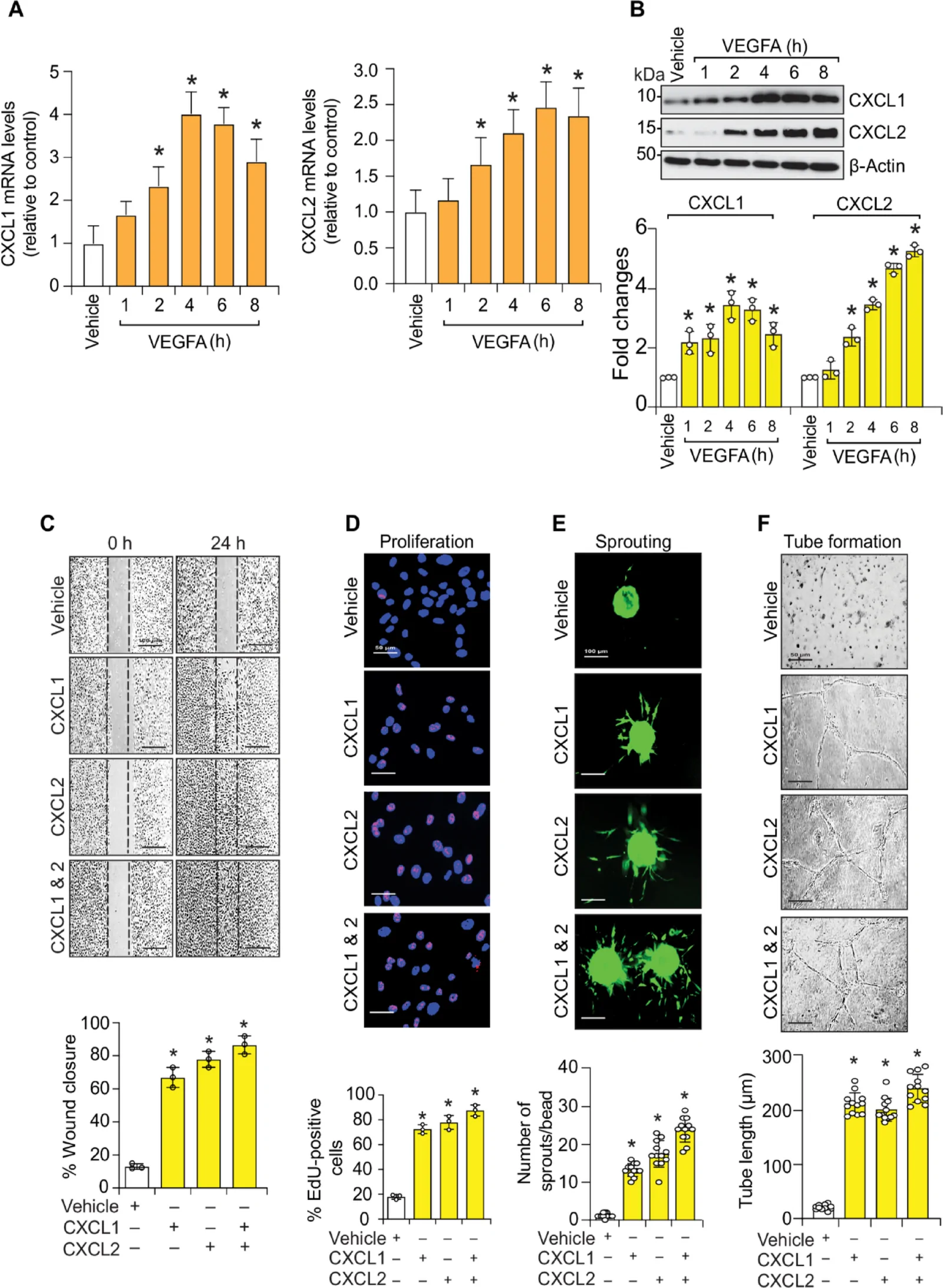
CXCL1/2 promotes mouse retinal microvascular endothelial cell migration, proliferation, sprouting and tube formation. **A** and **B** Quiescent MRMVECs were treated with vehicle or VEGFA (40 ng/ml) for the indicated time periods, and either mRNA was isolated or protein extracts were prepared. The mRNA and protein extracts were analyzed by qRT-PCR (**A**) and Western blotting (**B**) for CXCL1, CXCL2 and β-actin levels using their specific primers or antibodies, respectively. **C–F** Quiescent MRMVECs were treated with vehicle or recombinant CXCL1 (50 ng/ml) or CXCL2 (50 ng/ml) alone and in combination and subjected to migration (**C**), proliferation (**D**), sprouting (**E**) and tube formation (**F**) assays. The bar graphs represent quantitative analyses of 3 biological replicates, and the values are presented as Mean ± SD. *P < 0.05 vs. Vehicle. Scale bars in panels D and F are 50 μm and C and E are 100 μm

**Fig. 2:**
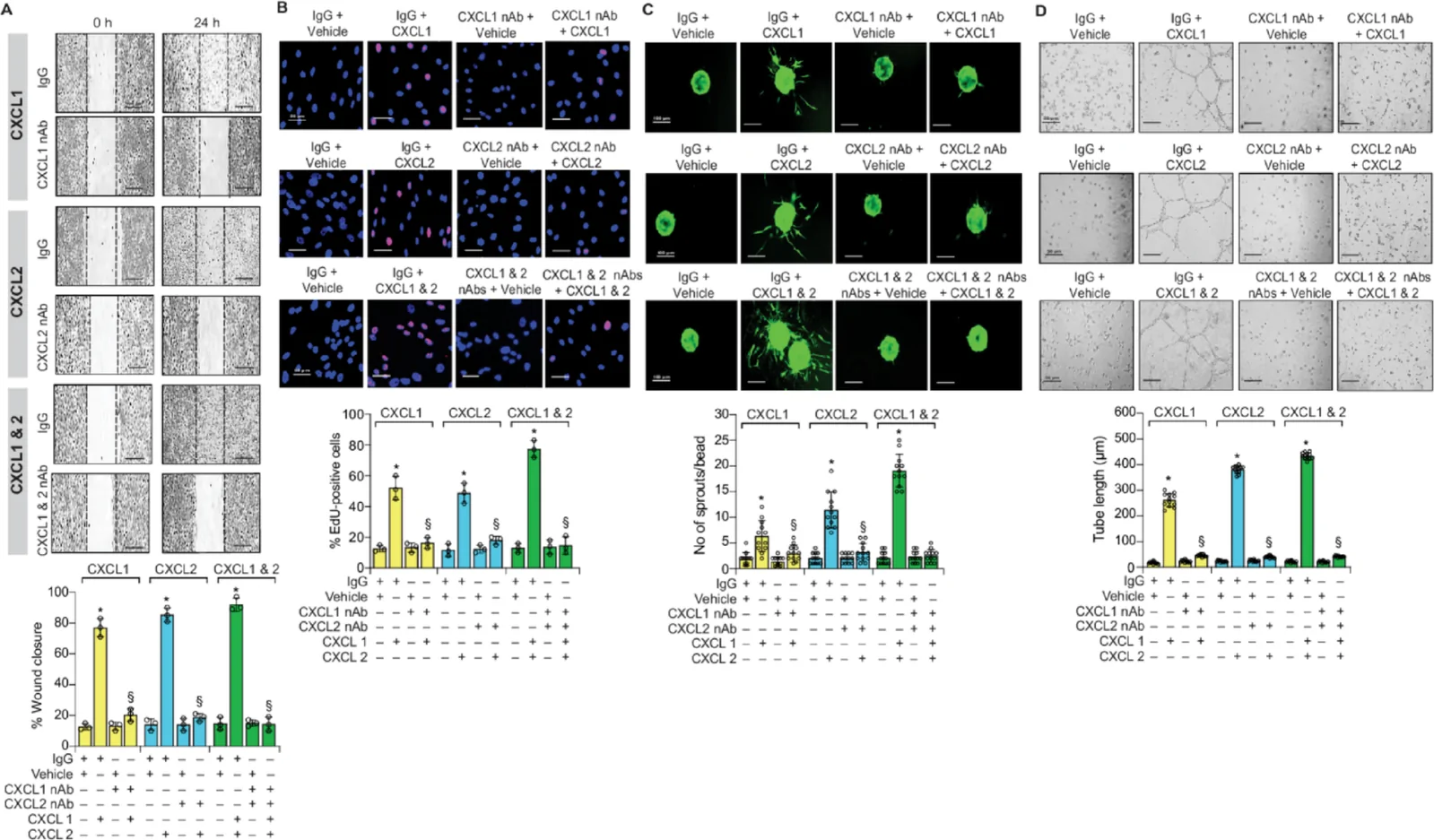
Blockade of CXCL1 and CXCL2 function by their neutralizing antibodies attenuates their pro-angiogenic effects in MRMVECs. **A–D** Quiescent MRMVECs were treated with vehicle or recombinant CXCL1 (50 ng/ml) or CXCL2 (50 ng/ml), alone or in combination in the presence or absence of their neutralizing Abs and subjected to migration (**A**), proliferation (**B**), sprouting (**C**) and tube formation (**D**) assays. The bar graphs represent quantitative analyses of 3 biological replicates, and the values are presented as Mean ± SD. *P < 0.05 vs. IgG + Vehicle; ^§^*P* < 0.05 vs. IgG + CXCL1 or IgG + CXCL2 or IgG + CXCL1 and CXCL2. Scale bars in panels B and D are 50 μm and A and C are 100 μm

**Fig. 3:**
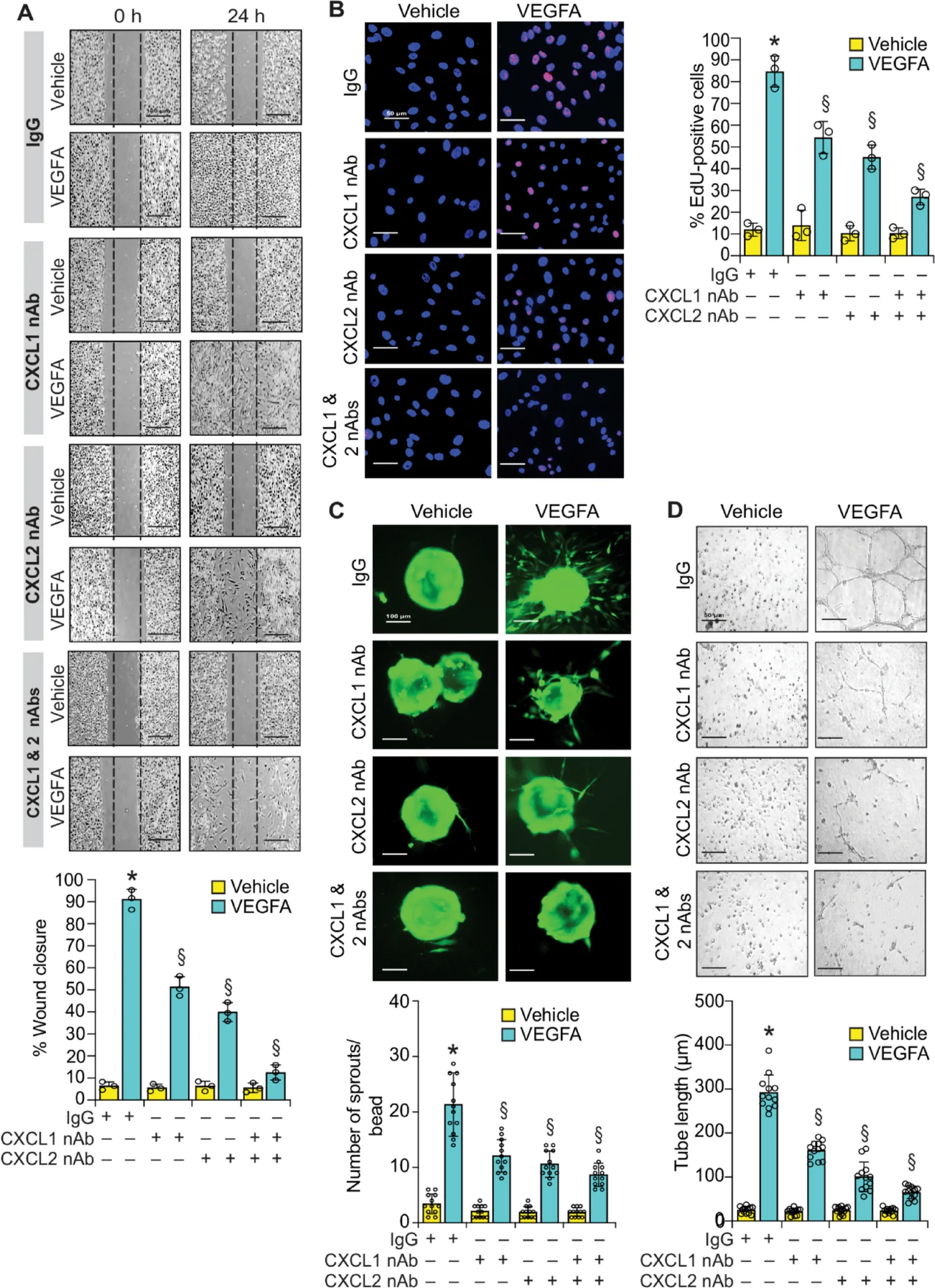
Blockade of CXCL1 and CXCL2 function by their neutralizing antibodies abrogates VEGFA-induced angiogenic effects in MRMVECs. **A–D** Quiescent MRMVECs were treated with vehicle or VEGFA (40 ng/ml) in the presence or absence of CXCL1 (1 µg/ml) or CXCL2 (0.4 µg/ml) neutralizing Abs, alone or in combination and subjected to migration (**A**), proliferation (**B**), sprouting (**C**) and tube formation (**D**) assays. The bar graphs represent quantitative analyses of 3 biological replicates, and the values are presented as Mean ± SD. *P < 0.05 vs. IgG + Vehicle; ^§^*P* < 0.05 vs. IgG + VEGFA. Scale bars in panels B and D are 50 μm and A and C are 100 μm

VEGFA plays a crucial role in the developmental and pathological angiogenesis [23, 24]. However, how VEGFA contributes to these phenomena at molecular level is not completely understood. Elevated levels of IL-8 have been reported in the vitreous humor of PDR patients, suggesting a potential role for IL-8 in pathological retinal angiogenesis [25, 26]. But the role of IL-8 in the modulation of retinal neovascularization has never been investigated. Mice express CXCL1 and CXCL2, the functional homologues of human IL-8. Therefore, in this study, we tested the interrelationship between VEGFA and CXCL1/2 in the regulation of pathological retinal angiogenesis using MRMVECs. We found that VEGFA induces the expression of both CXCL1 and CXCL2 at mRNA and protein levels in a time-dependent manner with maximum effects at 4 h and sustaining these effects thereafter (Fig. 1A, B). These molecules belong to a family of chemokines that appear to act as chemical messengers guiding endothelial cells to migrate, proliferate and organize into new vessels [14]. Based on these clues, we studied the role of CXCL1 and CXCL2 in VEGFA-induced angiogenic events. When added CXCL1 or CXCL2 individually to MRMVECs, each chemokine significantly enhanced the migration, proliferation, sprouting, and tube formation of these cells (Fig. 1C–F). When CXCL1 and CXCL2 were added together, no additive or synergistic effects were observed on these angiogenic events as compared to their individual effects (Fig. 1C–F). To determine the functional requirement of CXCL1 and CXCL2 for these responses, we next studied the effects of their neutralizing antibodies. Neutralization of CXCL1 or CXCL2 alone attenuated MRMVEC migration as evidenced by reduced wound closure compared to their respective controls (Fig. 2A). Simultaneous blockade of both CXCL1 and CXCL2 produced a more pronounced inhibition of MRMVEC migration, indicating that their effects were partially overlapping on endothelial motility (Fig. 2A). Similarly, neutralization of CXCL1 or CXCL2 alone blunted their effects on MRMVEC proliferation as measured by EdU incorporation (Fig. 2B). Combinatorial neutralization of both these chemokines almost completely abolished the MRMVEC proliferation revealing their cooperative role in endothelial cell growth (Fig. 2B). Sprouting of endothelial cells is a critical step in new blood vessel formation [27, 28]. While treatment with either chemokine robustly increased the number of sprouts per bead, this effect was significantly suppressed by their respective neutralizing Abs and nearly eliminated when both chemokines’ functions were blocked together (Fig. 2C). Finally, neutralization of CXCL1 or CXCL2 alone inhibited MRMVEC tube formation and tube length substantially and when both the chemokines were neutralized simultaneously the tube formation was blocked completely (Fig. 2D). Control IgGs had no effect on CXCL1 or CXCL2-induced MRMVEC migration, proliferation, sprouting or tube formation, confirming the specificity of their neutralizing antibodies. To determine whether these chemokines are required for VEGFA-induced angiogenic responses, we next studied the effects of CXCL1 and CXCL2 neutralizing antibodies on MRMVEC migration, proliferation, sprouting and tube formation. While neutralization of CXCL1 or CXCL2 alone reduced VEGFA-induced migration, proliferation, sprouting and tube formation of MRMVECs substantially, their combinatorial blockade completely suppressed these effects (Fig. 3A–D). These results indicate that VEGFA requires CXCL1 and CXCL2 to promote endothelial motility, proliferation, sprouting and tube formation.

### VEGFR2 and VEGFR3 via activation of ATF6-GRP78 signaling axis induce CXCL1/2 expression

**Fig. 4:**
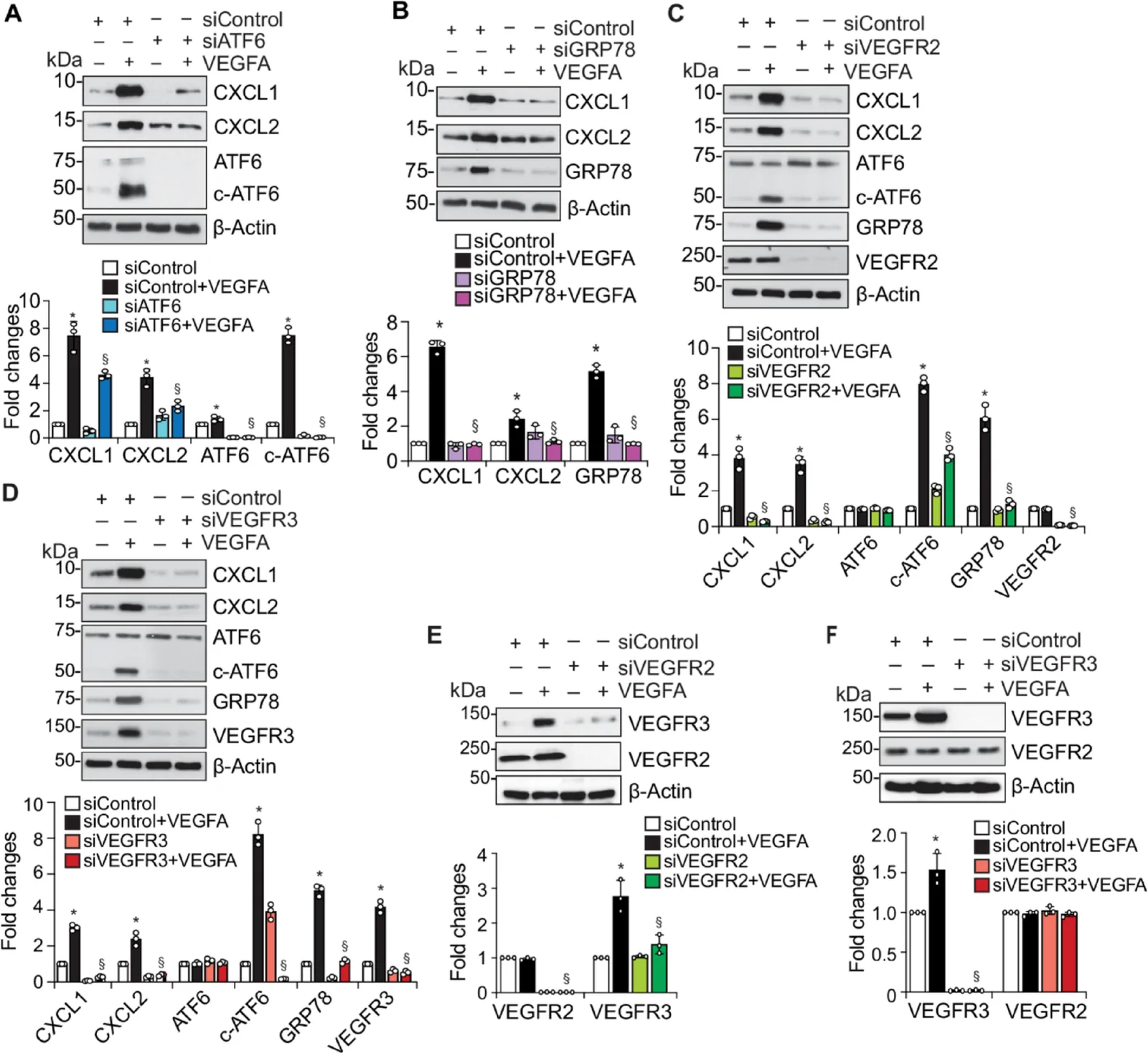
Both VEGFR2 and VEGFR3 are required for VEGFA-induced activation of ATF6-GRP78 signaling leading to CXCL1/2 expression in MRMVECs. **A** and **B** MRMVECs were transfected with siControl or siATF6 (**A**) or siGRP78 (**B**), treated with and without VEGFA (40 ng/ml) for 4 h and equal amounts of protein from control and each treatment were analyzed by Western blotting for the indicated proteins and the blots were normalized to β-actin. **C–F** MRMVECs were transfected with siControl or siVEGFR2 (**C** and **E**) or siVEGFR3 (**D** and **F**), treated with and without VEGFA (40 ng/ml) for 4 h and protein extracts were prepared and analyzed by Western blotting for the indicated proteins and the blots were normalized to β-actin. The bar graphs represent quantitative analyses of 3 biological replicates, and the values are presented as Mean ± SD. *P < 0.05 vs. siControl + Vehicle; ^§^*P* < 0.05 vs. siControl + VEGFA

Previously, we reported that the ATF6-GRP78 arm of the ER stress responsive signaling plays a role in retinal neovascularization [22]. Therefore, we tested the role of ATF6-GRP78 signaling in VEGFA-induced CXCL1 and CXCL2 expression. VEGFA triggered the cleavage and activation of ATF6, the ER stress-responsive transcription factor, along with upregulation of its downstream chaperone GRP78 (Fig. 4A, B). Depleting ATF6 levels markedly reduced VEGFA-induced CXCL1 and CXCL2 expression, demonstrating that ATF6 plays an important role in their induction (Fig. 4A). Similarly, siRNA-mediated depletion of GRP78 significantly blunted VEGFA-induced CXCL1 and CXCL2 expression (Fig. 4B). Together, these findings reveal that VEGFA engages ER stress-responsive signaling (ATF6-GRP78) to amplify inflammatory chemokine production, which in turn, drives aggressive endothelial angiogenic events. Previous reports showed that while VEGFA binds preferentially to VEGFR2, VEGFC and VEGFD bind to VEGFR3 in mediating angiogenesis and lymphangiogenesis, respectively [29, 30]. It was also shown that VEGFR2 interacts with VEGFR3 in enhancing angiogenesis [30]. We have shown that VEGFA induces VEGFR3 expression via VEGFR2 and that both the receptors were involved in the modulation of retinal angiogenesis [31]. Based on these findings, we now tested the role of VEGFR2 and VEGFR3 in VEGFA-induced CXCL1 and CXCL2 expression. Depletion of either VEGFR2 or VEGFR3 levels using their siRNAs attenuated VEGFA-induced ATF6 cleavage and GRP78 induction (Fig. 4C, D). Similarly, downregulation of VEGFR2 or VEGFR3 levels blocked VEGFA-induced CXCL1 and CXCL2 expression (Fig. 4C, D). In addition, while depletion of VEGFR2 levels negated VEGFA-induced VEGFR3 levels, downregulation of VEGFR3 levels had no effect on VEGFR2 levels (Fig. 4E, F). These observations suggest that VEGFR2-dependent VEGFR3 induction is required for VEGFA-induced ATF6-GRP78 signaling activation leading to CXCL1 and CXCL2 expression.

### VEGFR2 and VEGFR3 via activation of ATF6-GRP78 signaling axis leads to IL-8 induction in the mediation of VEGFA-induced angiogenic events in human retinal microvascular endothelial cells

**Fig. 5:**
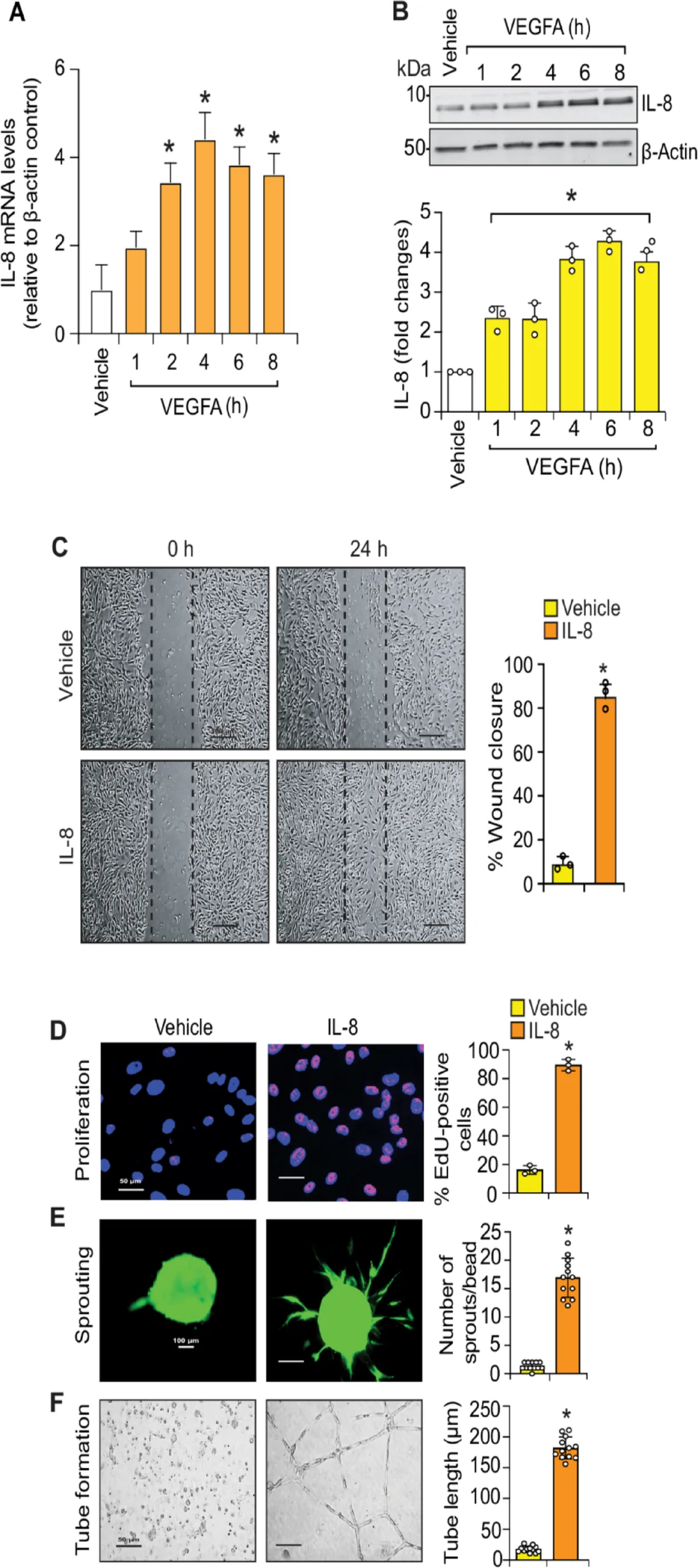
IL-8 promotes human retinal microvascular endothelial cell (HRMVEC) migration, proliferation, sprouting and tube formation. **A** and **B** Quiescent HRMVECs were treated with vehicle or VEGFA (40 ng/ml) for the indicated time periods, and either mRNA was isolated or protein extracts were prepared. The mRNA and protein extracts were analyzed by qRT-PCR (**A**) and Western blotting (**B**) for IL-8 and β-actin levels using their specific primers and antibodies, respectively. **C–F** Quiescent HRMVECs were treated with vehicle or recombinant IL-8 (100 ng/ml) and subjected to migration (**C**), proliferation (**D**), sprouting (**E**) and tube formation (**F**) assays. The bar graphs represent quantitative analyses of 3 biological replicates, and the values are presented as Mean ± SD. *P < 0.05 vs. Vehicle. Scale bars in panels D and F are 50 μm and C and E are 100 μm

**Fig. 6:**
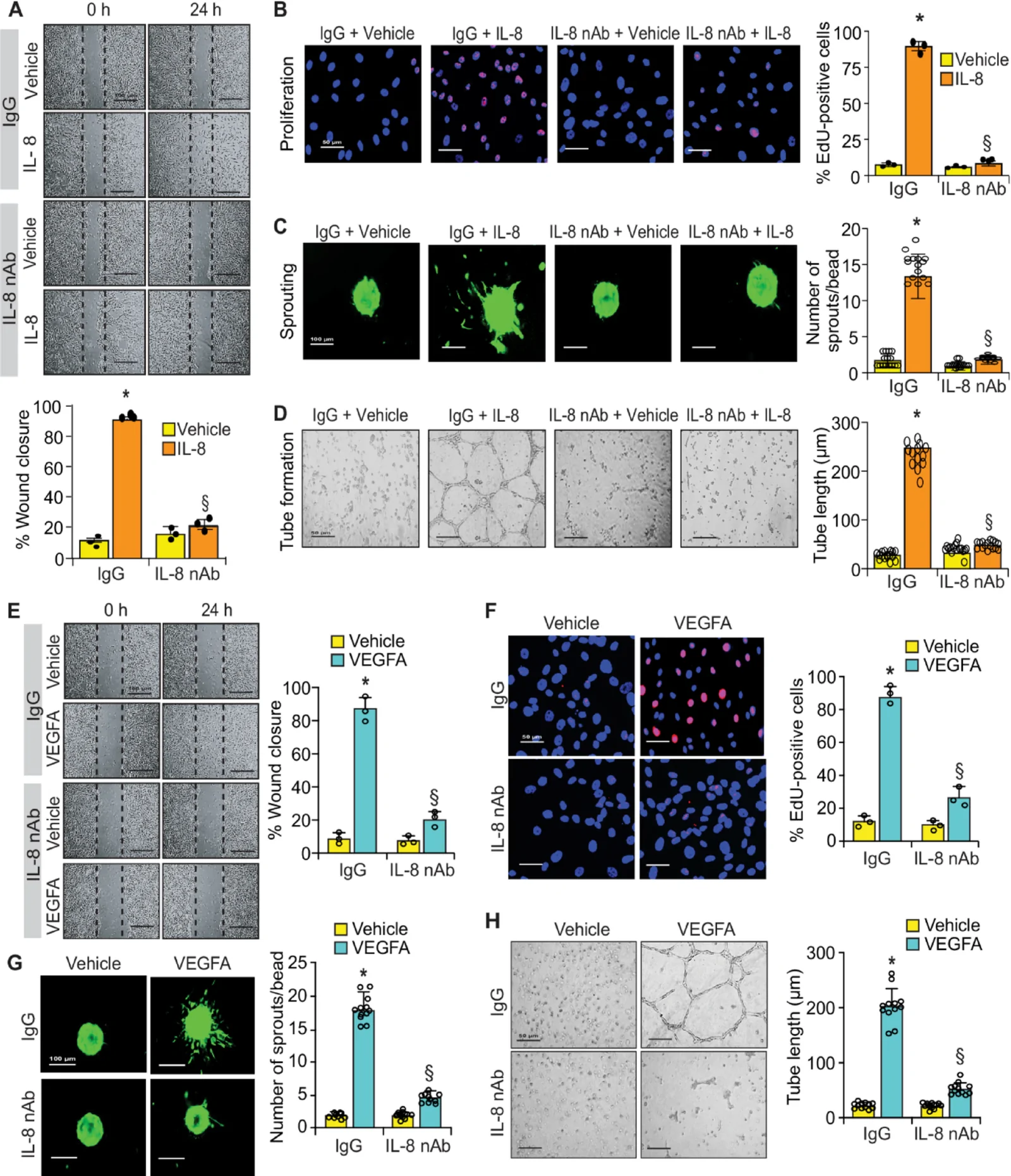
Blockade of IL-8 function by its neutralizing antibody abrogates IL-8 and VEGFA-induced angiogenic effects in HRMVECs. **A–D** Quiescent HRMVECs were treated with vehicle or recombinant IL-8 (100 ng/ml) in the presence or absence of IgG or IL-8 neutralizing antibodies (0.4 µg/ml) and subjected to migration (**A**), proliferation (**B**), sprouting (**C**) and tube formation (**D**) assays. **E**–**H** Quiescent HRMVECs were treated with vehicle or VEGFA (40 ng/ml) in the presence or absence of IgG or IL-8 neutralizing Abs (0.4 µg/ml) and subjected to migration (**E**), proliferation (**F**), sprouting (**G**) and tube formation (**H**) assays. The bar graphs represent quantitative analyses of 3 biological replicates, and the values are presented as Mean ± SD. *P < 0.05 vs. IgG + Vehicle; ^§^*P* < 0.05 vs. IgG + IL-8 or IgG + VEGFA. Scale bar in panels B, D, F and H are 50 μm and in panels A, C, E and G are 100 μm

**Fig. 7:**
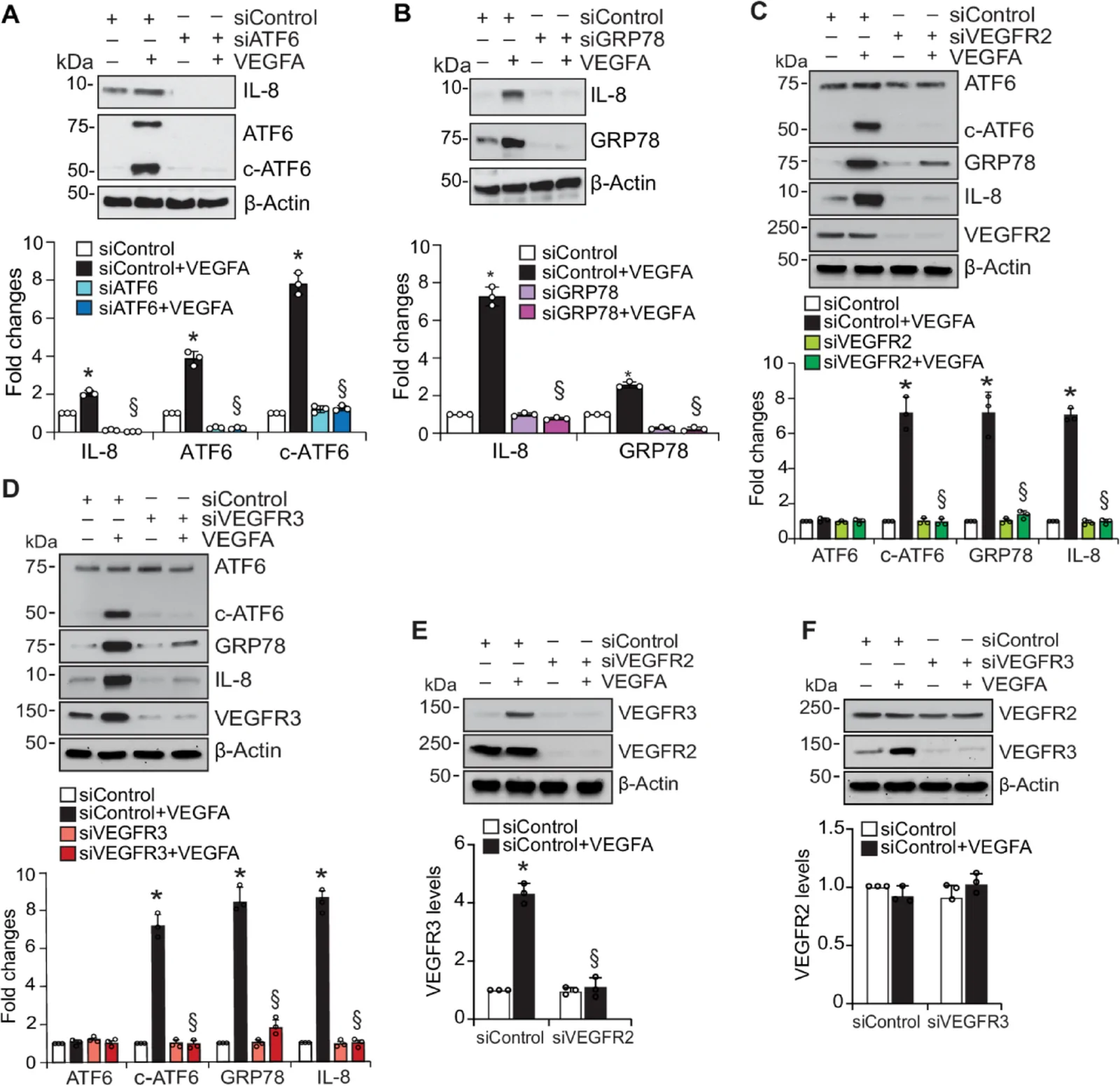
VEGFR2 and VEGFR3 are essential for VEGFA-driven activation of ATF6-GRP78 signaling and IL-8 expression in HRMVECs.** A** and **B** HRMVECs were transfected with siControl or siATF6 (**A**) or siGRP78 (**B**), treated with and without VEGFA (40 ng/ml) for 4 h and equal amounts of protein from control and each treatment were analyzed by Western blotting for the indicated proteins and the blots were normalized to β-actin. **C–F** HRMVECs were transfected with siControl or siVEGFR2 (**C** and **E**) or siVEGFR3 (**D** and **F**), treated with and without VEGFA (40 ng/ml) for 4 h and protein extracts were prepared and analyzed by Western blotting for the indicated proteins and the blots were normalized to β-actin. The bar graphs represent quantitative analyses of 3 biological replicates, and the values are presented as Mean ± SD. *P < 0.05 vs. siControl + Vehicle; ^§^*P* < 0.05 vs. siControl + VEGFA

Because IL-8 (CXCL8) is the human homolog of murine CXCL1/2 [25, 26], we next asked whether a similar signaling cascade operates in HRMVECs. Consistent with its effects on CXCL1 and CXCL2 expression in MRMVECs, VEGFA also triggered a robust and time-dependent increase in IL-8 expression at mRNA and protein levels in HRMVECs (Fig. 5A, B). The IL-8 expression in HRMVECs followed a kinetic pattern of CXCL1/2 upregulation in MRMVECs, suggesting that VEGFA engages a conserved chemokine program across the species. Functionally, IL-8 alone was sufficient to promote the hallmark angiogenic events such as HRMVEC migration, proliferation, sprouting and tube formation (Fig. 5C–F). These findings mirror the CXCL1/2-driven endothelial cell responses observed in mouse, reinforcing the idea that chemokine signaling acts as a key amplifier of VEGFA-induced angiogenesis. To determine whether IL-8 is merely an associated factor or an essential mediator of VEGFA-driven angiogenesis, we next examined the effects of IL-8 neutralization on both IL-8 and VEGFA-induced endothelial angiogenic responses. Neutralizing IL-8 antibody markedly inhibited IL-8-induced HRMVEC migration, proliferation, sprouting and tube formation (Fig. 6A–D). Neutralization of IL-8 also inhibited VEGFA-induced HRMVEC migration, proliferation, sprouting and tube formation (Fig. 6E–H). Together, these data establish that IL-8 is not simply a parallel angiogenic factor, but a central downstream effector of VEGFA-driven angiogenic responses in HRMVECs. Mechanistically, VEGFA-induced IL-8 expression required activation of the same ER stress-responsive signaling identified earlier. Depleting ATF6 levels by its siRNA significantly reduced VEGFA-induced IL-8 expression along with suppression of ATF6 cleavage to its active form (Fig. 7A). Similarly, siRNA-mediated depletion of GRP78 levels markedly attenuated VEGFA-induced IL-8 induction (Fig. 7B). These results demonstrate that the ATF6-GRP78 arm of the ER stress responsive signaling is required for VEGFA-induced IL-8 expression mediating migration, proliferation, sprouting and tube formation of HRMVECs. To define how VEGF receptor signaling mediates IL-8 production, we next examined the relative contributions of VEGFR2 and VEGFR3. Depleting either VEGFR2 or VEGFR3 levels using their siRNAs significantly attenuated VEGFA-induced ATF6 activation and GRP78 expression leading to a marked reduction in IL-8 levels (Fig. 7C, D). In addition, depletion of VEGFR2 levels blunted VEGFA-induced VEGFR3 expression, whereas downregulation of VEGFR3 levels had no effect on VEGFR2 levels (Fig. 7E, F). These findings indicate that VEGFR2-dependent VEGFR3 induction is required for activation of VEGFA-induced ATF6-GRP78 signaling mediating IL-8 expression.

### CXCR2 mediates CXCL1/2 and IL-8-induced mouse and human retinal microvascular endothelial cell angiogenic responses, respectively

**Fig. 8:**
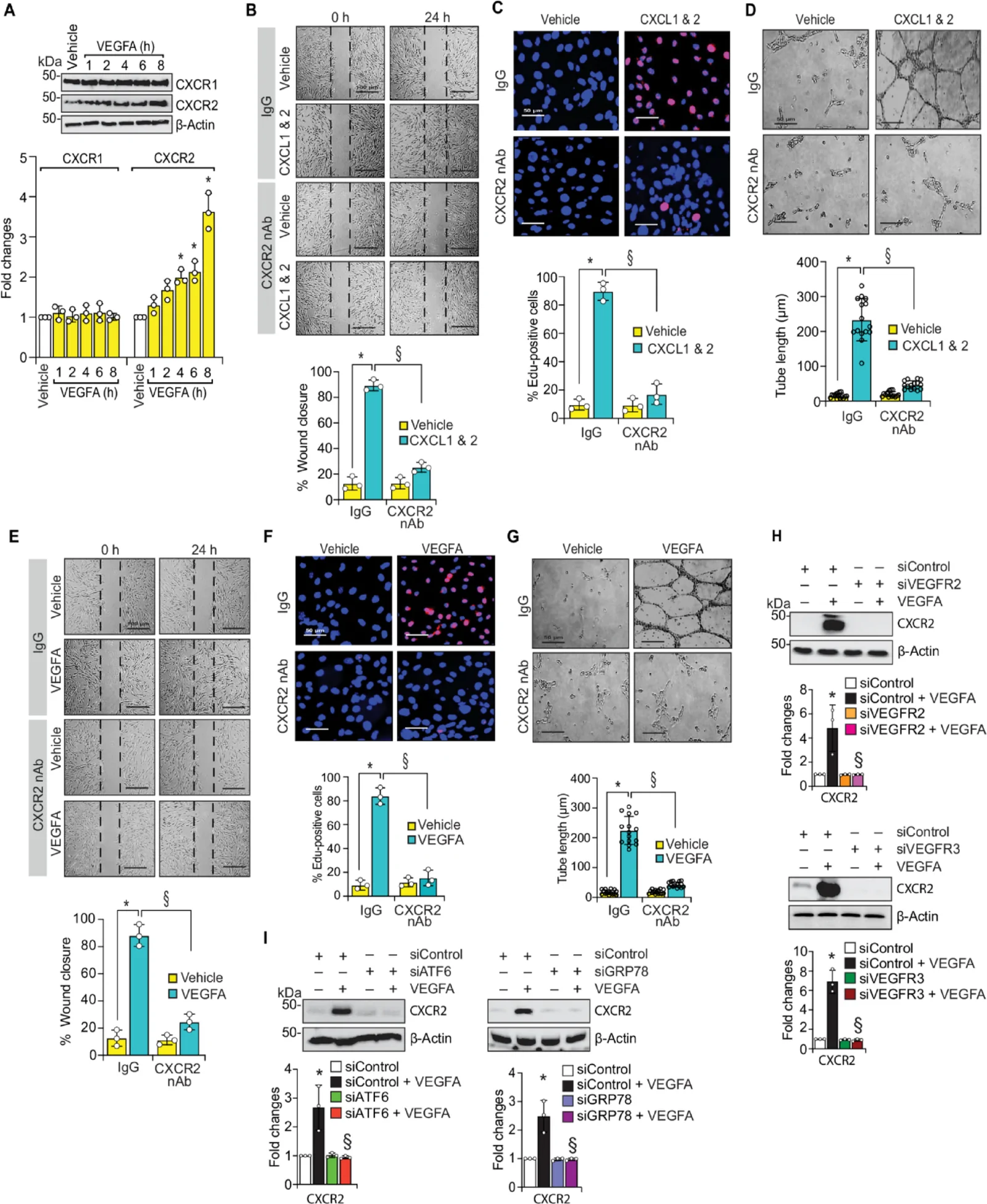
CXCR2 mediates CXCL1/2 or VEGFA-induced MRMVEC angiogenic responses. **A** Quiescent MRMVECs were treated with vehicle or VEGFA (40 ng/ml) for the indicated time periods and protein extracts were prepared and analyzed by Western blotting for CXCR1, CXCR2, and β-actin levels using their specific antibodies. **B–G** Quiescent MRMVECs were treated with vehicle or recombinant CXCL1/2 (50 ng each/ml) or VEGFA (40 ng/ml) in the presence or absence of CXCR2 neutralizing Abs (3 µg/ml) and were subjected to migration (**B**, **E**), proliferation (**C**, **F**), or tube formation (**D**, **G**) assays. **H**–**I** MRMVECs were transfected with siControl, siVEGFR2 or siVEGFR3 (**H**) and siATF6 or siGRP78 (**I**), treated with and without VEGFA (40 ng/ml) for 4 h and protein extracts were prepared and analyzed by Western blotting for CXCR2 levels. The blot was normalized to β-actin. The bar graphs represent quantitative analysis of 3 biological replicates. The values are presented as Mean ± SD. *P < 0.05 vs. IgG + Vehicle or siControl + Vehicle; ^§^*P* < 0.05 vs. IgG + CXCL1/2 or VEGFA or siControl + VEGFA. Scale bar in panels C, D, F and G are 50 μm and in panels B and E are 100 μm

**Fig. 9:**
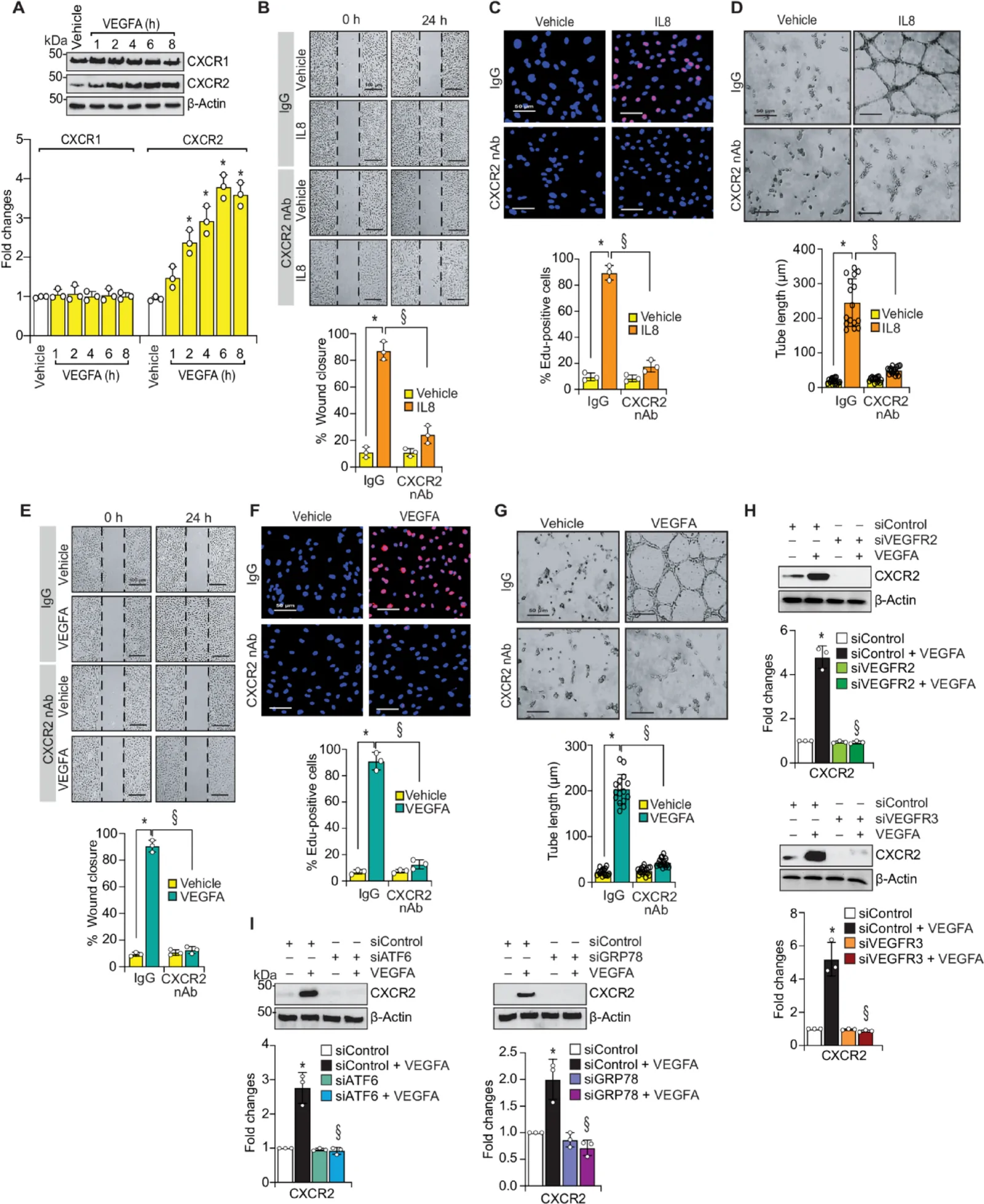
CXCR2 mediates IL-8 or VEGFA-induced HRMVEC angiogenic responses. **A** Quiescent HRMVECs were treated with vehicle or VEGFA (40 ng/ml) for the indicated time periods and protein extracts were prepared and analyzed by Western blotting for CXCR1, CXCR2, and β-actin levels using their specific antibodies. **B**–**G** Quiescent HRMVECs were treated with vehicle or recombinant IL-8 (100 ng/ml) or VEGFA (40 ng/ml) in the presence or absence of CXCR2 neutralizing Abs (3 µg/ml) and subjected to migration (**B**, **E**), proliferation (**C**, **F**) or tube formation (**D**, **G**) assays. **H–I** HRMVECs were transfected with siControl, siVEGFR2 or siVEGFR3 (**H**) and siATF6 or siGRP78 (**I**), treated with and without VEGFA (40 ng/ml) for 4 h and protein extracts were prepared and analyzed by Western blotting for CXCR2 levels. The blot was normalized to β-actin. The bar graphs represent quantitative analysis of 3 biological replicates. The values are presented as Mean ± SD. *P < 0.05 vs. IgG + Vehicle or siControl + Vehicle; ^§^*P* < 0.05 vs. IgG + IL-8 or VEGFA or siControl + VEGFA. Scale bar in panels C, D, F and G are 50 μm and in panels B and E are 100 μm

As CXCL1/2 in mouse and IL-8 in humans bind to CXCR1/2 in exerting their cellular effects, we next studied the effects of VEGFA on their expression. VEGFA without having no effect on CXCR1 level, induced CXCR2 expression in a time-dependent manner both in MRMVECs and HRMVECs (Figs. 8A and 9A). Based on these observations, we next investigated the role of CXCR2 in the angiogenic responses of these cells. Neutralization of CXCR2 inhibited CXCL1/2 and IL-8-induced migration, proliferation and tube formation of MRMVECs and HRMVECs, respectively (Figs. 8B–D and 9B–D). Neutralizing CXCR2 Abs also blunted VEGFA-induced migration, proliferation and tube formation of MRMVECs and HRMVECs (Figs. 8E–G and 9E–G). These observations clearly demonstrate that CXCR2 mediates CXCL1/2 and IL-8-induced endothelial cell angiogenic responses downstream to VEGFA both in mouse and human retinal microvascular endothelial cells. Regarding the mechanisms of CXCR2 expression, downregulation of either VEGFR2 or VEGFR3 levels by their siRNAs attenuated its induction by VEGFA both in MRMVECs and HRMVECs (Figs. 8H and 9H). Similarly, siRNA-mediated depletion of ATF6 or GRP78 levels inhibited VEGFA-induced CXCR2 expression in MRMVECs and HRMVECs (Figs. 8I and 9I).

### CXCL1/2-CXCR2 axis drives pathological retinal neovascularization

**Fig. 10:**
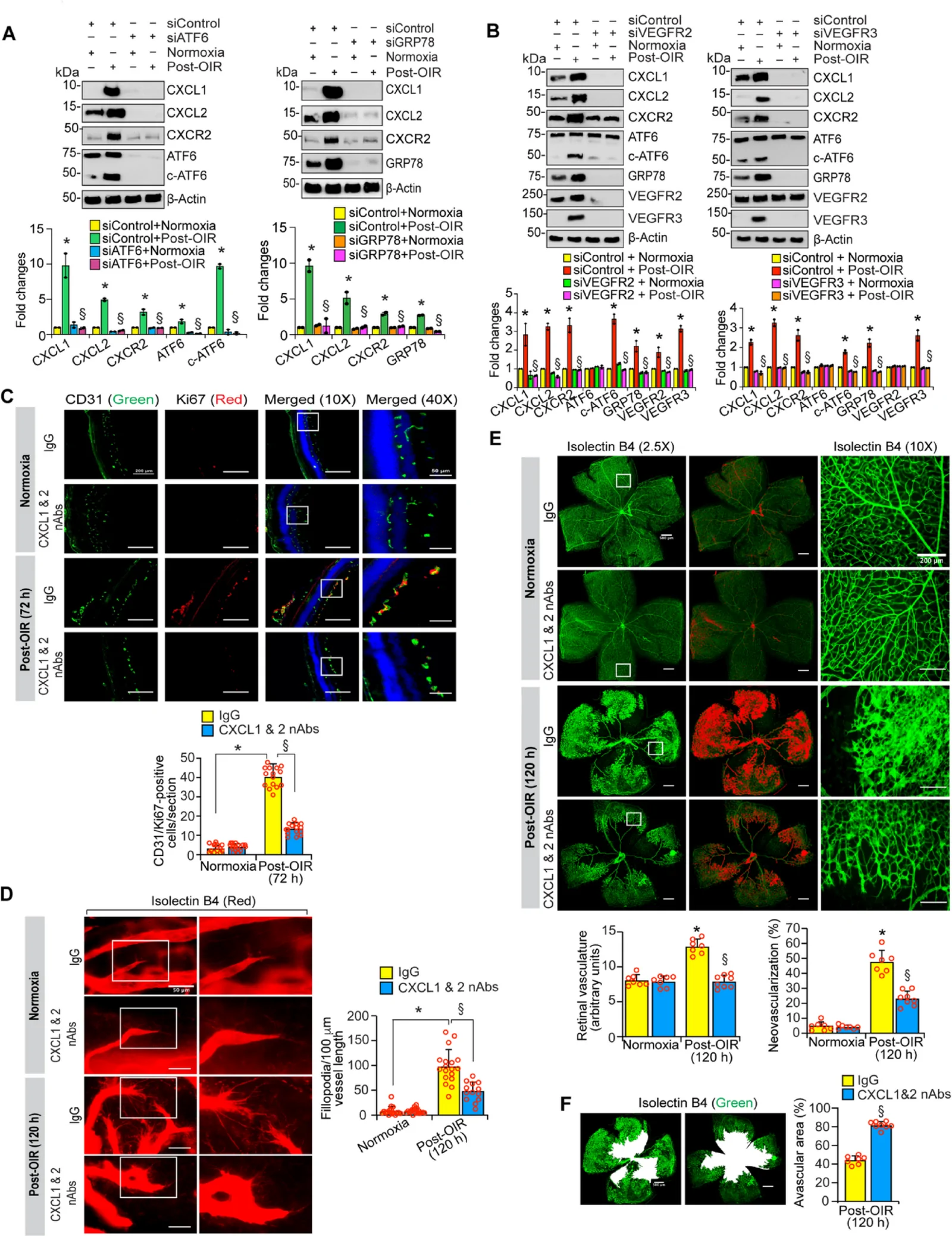
Activation of VEGFR2-VEGFR3-ATF6-GRP78 signaling axis is required for OIR-induced CXCL1/2 expression leading to pathological retinal angiogenesis. **A** and **B** Retinal extracts of normoxic and 72-h post OIR mouse pups that received siControl, siATF6 or siGRP78 (**A**) or siVEGFR2 or siVEGFR3 (**B**) are analyzed by Western blotting for the indicated proteins using their specific antibodies and the blots were normalized to β-actin. **C** Immunofluorescence staining for CD31 and ki67 of retinal cross sections from normoxic and 72-h of post-OIR pups that received control IgG or CXCL1/2 neutralizing Abs to show the retinal EC proliferation. The square areas marked in the 3rd column were shown at 40X magnification in the immediate right column. The retinal EC proliferation was measured by counting CD31 and Ki67-positive cells in each section and shown in the bar graph. **D** All the conditions were the same as in panel C except that eyes were enucleated from normoxic and 120-h of post-OIR pups, fixed, retinas isolated, stained with isolectin B4, flat mounts were made and were examined for filopodia-like protrusions at 40X magnification. **E** and **F** All the conditions were the same as in panel C except that the flat mounts were analyzed for neovascularization (**E**) and avascular area (**F**). The neovascularization was highlighted in pseudo red color in the 2nd column of panel E. The marked square areas in the 1st column of panel E were shown at 10X magnification in the far-right column. The retinal vasculature, neovascularization and avascular area were shown in the bar graph. The data are presented as Mean ± SD of 7 biological replicates. *P < 0.05 vs. siControl + Normoxia or IgG + Normoxia; ^§^*P* < 0.05 vs. siControl + post-OIR or IgG + post-OIR. Scale bars in panel C are 200 μm (10X) and 50 μm (40X). Scale bar in panel D is 50 μm. Scale bars in panel E and F are 500 μm (2.5X) and 200 μm (10X)

**Fig. 11:**
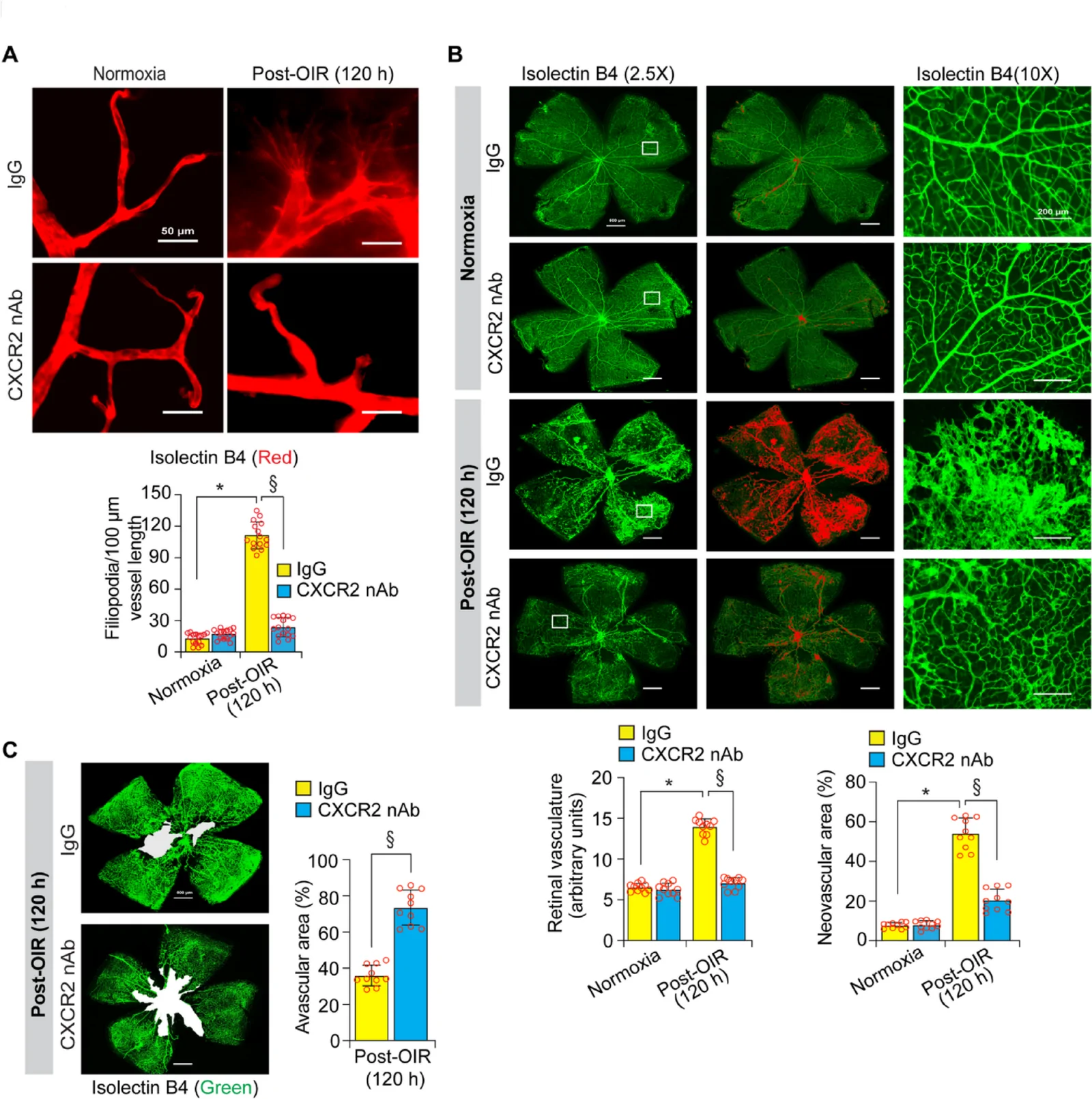
CXCR2 drives pathological retinal neovascularization. **A** Normoxic and hyperoxic mouse pups were injected intravitreally with 1 µg/0.5 µl/eye of IgG or CXCR2 neutralizing Abs at P11 and P12. The eyes were enucleated from normoxic and 120-h of post-OIR pups, fixed, retinas isolated, stained with isolectin B4, flat mounts were made and were examined for filopodia-like protrusions at 40X magnification. **B** and **C** All the conditions were the same as in panel A except that flat mounts were made and analyzed for neovascularization (**B**) and avascular area (**C**). The neovascularization was highlighted in pseudo red color in the 2nd column of panel B. The marked square areas in the 1st column of panel B were shown at 10X magnification in the far-right column. The retinal vasculature, neovascularization and avascular area were shown in the bar graph. The data were presented as Mean ± SD of 7 biological replicates. *P < 0.05 vs. IgG + normoxia; ^§^*P* < 0.05 vs. IgG + post-OIR. Scale bar in panel A is 50 μm. Scale bars in panels B and C are 500 μm (2.5X) and 200 μm (10X)

**Fig. 12:**
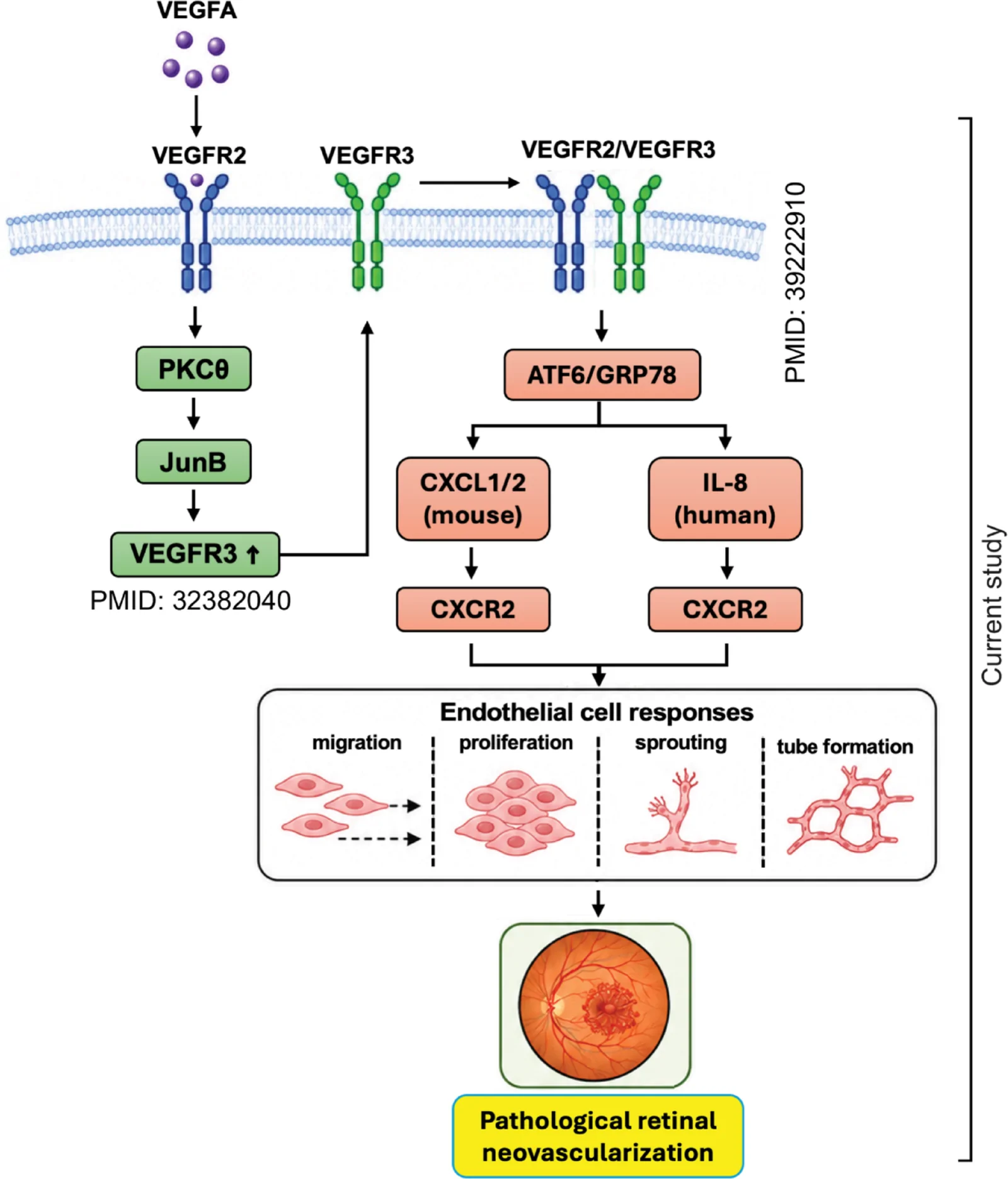
Schematic diagram depicting ATF6-GRP78 signaling in the expression of CXCL1/2 and or IL-8 in the modulation of retinal neovascularization

The in vitro findings demonstrate that VEGFA induces CXCL1/2, IL-8 and CXCR2 through activation of the ATF6-GRP78 arm of the ER stress-response signaling in MRMVECs and HRMVECs. To determine whether the same signaling events also operate in vivo, we next examined the role of ATF6-GRP78-CXCL1/2-CXCR2 signaling in OIR model, a well-established animal model of pathological retinal neovascularization [32]. Mouse pups were kept in hyperoxia (75% O_2_) chamber from P7 to P12 and returned to room air at P12 to develop relative hypoxia. Mouse pups from the same litter kept in normoxia were used as controls. The eyes from normoxia and 72-h post-OIR pups were enucleated, retinas were isolated and protein extracts were prepared and analyzed by Western blotting for ATF6, GRP78, CXCL1/2 and CXCR2 levels. The retinas from 72-h post-OIR pups showed activation of ATF6 as measured by its cleavage and induction of GRP78, CXCL1/2 and CXCR2 expression as compared to retinas from normoxic pups (Fig. 10A). Depletion of ATF6 or GRP78 levels using their siRNAs blunted the post-OIR-induced CXCL1/2 and CXCR2 expression, confirming that ATF6-GRP78 signaling axis is required for the induction of these chemokines and their receptor CXCR2 in vivo. In addition, siRNA-mediated depletion of ATF6 levels suppressed post-OIR-induced GRP78 expression, suggesting that GRP78 acts downstream to ATF6. Besides, siRNA-mediated downregulation of VEGFR2 or VEGFR3 levels also inhibited post-OIR-induced ATF6 cleavage and GRP78, CXCL1/2 and CXCR2 expression (Fig. 10B). In addition, while depletion of VEGFR3 levels had no effect on VEGFR2 levels, downregulation of VEGFR2 levels negated post-OIR-induced VEGFR3 levels (Fig. 10B). All these findings reveal that VEGFR2-dependent VEGFR3 expression leading to ATF6 activation and GRP78 induction plays a role in post-OIR-induced CXCL1/2 and CXCR2 expression. Furthermore, coimmunofluorescence staining of retinal cross sections from 72-h post-OIR mouse pups for Ki67 and CD31 showed increased EC proliferation as compared to retinas of normoxic mouse pups and blockade of CXCL1/2 function using their neutralizing Abs attenuated this effect (Fig. 10C). To measure in vivo sprouting, the retinal flat mounts of normoxic and 120-h post-OIR mouse pups were stained with isolectin B4 and observed for filopodia-like protrusions under fluorescence microscope. Increased filopodia-like structures were observed in 120-h post-OIR mouse pups’ retinas as compared to normoxic mouse pups’ retinas and neutralizing CXCL1/2 or CXCR2 Abs completely suppressed this effect (Figs. 10D and 11A). Together, these findings demonstrate that CXCL1/2 and CXCR2 are required for retinal endothelial proliferation and sprouting. To determine whether these cellular effects translate into global changes in retinal vascular remodeling, we next examined the whole-mount retinal vasculature. Under normoxic conditions, retinal vascular organization was comparable between control IgG and CXCL1/2 or CXCR2 nAb-treated mouse pups indicating that blockade of CXCL1/2 or CXCR2 function does not disrupt normal retinal vasculature (Figs. 10E and 11B). In contrast, 120-h post-OIR retinas from control IgG-treated mouse pups exhibited extensive pathological retinal neovascularization, characterized by dense and disorganized vascular networks (Figs. 10E and 11B). Strikingly, CXCL1/2 or CXCR2 nAb-treated 120-h post-OIR retinas showed a significant reduction in the overall pathological retinal vascular density and neovascular area as visualized by isolectin B4 staining (Figs. 10E and 11B). Consistent with suppression of excessive neovascular growth, CXCL1/2 or CXCR2 nAb-treated mouse pups’ retinas showed an increased avascular area during the post-OIR phase compared to control IgG group (Figs. 10F and 11C). Since blocking CXCL1/2 or their receptor CXCR2 function using their neutralizing Abs had no effect on normal vasculature, the increased avascular area under these conditions in post-OIR retinas infers their role only in pathological retinal angiogenesis but not in physiological angiogenesis. However, a role for CXCL1/2-CXCR2 axis in the reparative angiogenesis after OIR can also be possible, in which case, inhibition of this signaling may contribute to increased avascular area at least to some level. Together, as shown in Fig. 12, these findings demonstrate that CXCL1/2 and their receptor CXCR2 are important regulators of pathological but not physiological retinal angiogenesis, and that their functional suppression effectively limits abnormal vascular remodeling at the tissue level.

## Discussion

Retinal neovascularization is a pathological hallmark of the vision-threatening retinal ischemic diseases such as PDR, ROP, and AMD [2, 3, 33]. VEGF is the central molecular driver of retinal neovascularization and plays a pivotal role in retinal ischemic diseases [23, 24]. Although VEGF has been shown as the central molecule of abnormal retinal angiogenesis, clinical studies have revealed that VEGF inhibition alone is insufficient to suppress these vision-threatening retinal ischemic diseases fully [34–36]. Partial responses, resistance, and recurrence are the major challenges that need to be resolved in VEGF-targeted therapies [34–36]. Therefore, further investigations are required to identify alternative targets to develop therapies to cure retinal ischemic diseases. To this end, we have previously demonstrated that the ATF6-GRP78 arm of the ER stress signaling downstream to VEGFR2 and VEGFR3 by releasing β-catenin from adherens junctions and its interaction with STAT3 mediates VEGFA/OIR-induced retinal neovascularization [22]. In the present study, we report that ELR⁺ (glutamic acid, leucine and arginine) CXC (cysteine-X-cysteine) chemokines CXCL1/2 and IL-8 (CXCL8) act as downstream regulators of VEGFR2-VEGFR3-ATF6-GRP78 signaling axis in mice and humans, respectively, mediating VEGFA/OIR-induced retinal neovascularization. We found that VEGFA induces CXCL1/2 expression both at mRNA and protein levels in MRMVECs in a time-dependent manner. CXCL1/2, while alone were able to trigger MRMVEC migration, proliferation, sprouting and tube formation substantially, in combination, they showed a little enhanced effect. Blockade of CXCL1/2 function using their neutralizing Abs blunted CXCL1/2-induced angiogenic responses of MRMVECs, alone or in a combinatorial manner. Besides, CXCL1/2 neutralizing Abs also blocked VEGFA-induced MRMVEC migration, proliferation, sprouting, and tube formation. In line with these observations, OIR induced CXCL1/2 expression in the mouse retina, and blockade of their function with their neutralizing Abs inhibited OIR-induced retinal neovascularization. Supporting these observations, VEGFA also induced CXCL1/2 receptor CXCR2 expression in MRMVECs in VEGFR2-VEGFR3-ATF6-GRP78-dependent manner and inhibition of its function by its neutralizing Abs suppressed the migration, proliferation and tube formation of these cells triggered by CXCL1/2 or VEGFA. Similarly, OIR induced CXCR2 expression in VEGFR2-VEGFR3-ATF6-GRP78-dependent manner and blockade of its function by its neutralizing Abs negated OIR-induced retinal neovascularization. As CXCL1/2 and their receptor CXCR2 were induced in MRMVECs in response to VEGFA, their expression in the mouse retina by OIR was likely to be occurred in ECs rather than other cell types such as pigment epithelial cells or photoreceptor cells. Towards the translational relevance, we found that VEGFA induces IL-8 and its receptor CXCR2 expression in HRMVECs mediating their migration, proliferation, sprouting and tube formation, and neutralizing IL-8 or CXCR2 Abs suppressed these effects.

CXCL1/2 are ELR⁺ CXC chemokines that primarily signal through the CXCR2 receptor, which is expressed in endothelial cells, neutrophils, and retinal glial cells [37, 38]. Both chemokines possess strong pro-angiogenic properties and are upregulated under hypoxic and inflammatory conditions [14, 39]. Increased CXCL1/2 expression in OIR mouse model correlates with pathological retinal angiogenesis suggesting a role for them in ischemia-induced vascular remodeling [39]. Mechanistically, CXCL1/2 have been shown to promote endothelial cell migration, proliferation and tube formation via activation of several signaling events such as ERK1/2, PI3K-AKT, and NFκB [39, 40]. In addition, CXCL1/2 by recruitment of neutrophils and amplifying retinal inflammation through the release of reactive oxygen species and proteases have been reported to enhance the angiogenic signaling events [41]. Importantly, blockade of CXCR2 signaling has been shown to reduce pathological neovascular tufts in murine OIR model, highlighting the functional relevance of the CXCL1/2-CXCR2 axis in retinal neovascularization [40]. Supporting these earlier reports, our present observations clearly show a role for CXCL1/2-CXCR2 in VEGFA/OIR-induced retinal neovascularization in mice. HIF1α mediates hypoxia-induced VEGF expression, which is well established [42, 43]. In addition, our findings show that both VEGFA and OIR-induced CXCL1/2 expression exhibits a requirement for activation of the same signaling events. So, it is most likely that OIR-induced CXCL1/2 expression is dependent on VEGFA and thereby by HIF1α. Thus, HIF1α may be expected to regulate CXCL1/2 and CXCR2.

IL-8 (CXCL8), the human orthologue of mouse CXCL1/2, is one of the most potent ELR⁺ CXC chemokines with well-established pro-angiogenic activity [25]. Previous studies have shown that IL-8 levels were increased in the vitreous and aqueous humor of patients with PDR and other neovascular retinal disorders [25, 26]. Retinal endothelial cells, Müller glia, and infiltrating immune cells are the major sources of IL-8 in the ischemic retina [25]. More intriguingly, IL-8 promotes retinal angiogenesis both directly by stimulating endothelial cell survival and migration through CXCR1 and CXCR2 and indirectly by synergizing with VEGF signaling [44]. Notably, IL-8 can induce VEGF expression and sustain angiogenesis even under conditions where VEGF signaling is therapeutically suppressed, potentially contributing to anti-VEGF resistance observed in some patients with PDR [44]. Furthermore, IL-8 enhances vascular permeability and leukostasis exacerbating retinal edema and inflammation, key drivers of disease progression [45–47]. Besides these observations, our findings reveal that IL-8 and its receptor CXCR2 are also required for VEGFA-induced HRMVEC migration, proliferation and tube formation, the events that are essential for retinal neovascularization in humans as well. However, further validation of IL-8 role in human retinal neovascularization may require additional studies using IL-8 humanized mouse model, which is a limitation.

Towards exploring the mechanisms by which VEGFA regulates CXCL1/2, IL-8 and CXCR2 expression in mice and humans, in view of our recent report [22], we tested the involvement of the ATF6-GRP78 arm of the ER stress signaling. Depletion of ATF6 or GRP78 levels blocked VEGFA-induced CXCL1/2 and CXCR2 expression in MRMVECs and IL-8 and CXCR2 induction in HRMVECs in vitro and OIR-induced CXCL1/2 and CXCR2 expression in the mouse retina in vivo. Thus, our findings demonstrate that CXCL1/2 and their human orthologue IL-8 along with their receptor CXCR2 as critical downstream regulators of ATF6-GRP78 signaling mediating VEGFA/OIR-induced retinal neovascularization in mice and humans, respectively. These findings are mechanistically supported by the accumulating evidence implicating that ER stress-responsive ATF6-GRP78 signaling as an upstream driver of inflammatory angiogenesis [48–50]. Hypoxia, a central pathological stimulus in ischemic retinopathies, induces ER stress through impaired protein folding and redox imbalance resulting in dissociation of ATF6 from GRP78 and its proteolytic activation in the Golgi leading to its nuclear translocation [48–50]. Nuclear ATF6 induces a transcriptional program that extends beyond proteostasis to trigger the production of inflammatory and angiogenic mediators [51, 52]. Multiple studies demonstrated that ER stress enhances the expression of CXCL1/2 and IL-8 via ATF6-dependent mechanisms, either directly or through a crosstalk with the NFκB or MAPK signaling pathway [53, 54]. ATF6 has been shown to cooperate with NFκB at promoter and enhancer regions of inflammatory genes amplifying chemokine transcription under hypoxia and oxidative stress conditions [53]. The findings that CXCL1/2 and IL-8 promoters contain canonical NFκB and AP-1 binding elements and ATF6 potentiates the expression of these chemokines may suggest a link between NFκB, AP-1 and ATF6 in chemokine induction by hypoxic retina [55]. Previous studies from other laboratories have reported that CXCL1/2 and IL-8 signaling acts upstream of VEGF in mediating angiogenic responses [56, 57]. Furthermore, these chemokines promote recruitment and activation of CXCR2-expressing myeloid cells, a major source of VEGFA in ischemic retina that directly enhances endothelial migration, proliferation, and survival [8, 56, 57]. CXCR2 signaling also activates ERK1/2, PI3K/AKT, and STAT3 pathways in parallel with VEGFA signaling to amplify angiogenic output [8, 56]. In the present study, we show that CXCL1/2 and IL-8 act downstream to ATF6-GRP78 signaling in the mediation of VEGFA/OIR-induced retinal angiogenesis. Thus, ATF6-GRP78-driven upregulation of CXCL1/2 or IL-8 along with their receptor CXCR2 may constitute a feed-forward inflammatory loop that enhances VEGFA bioavailability and endothelial responsiveness, rather than functioning as a parallel pathway. Consistent with the role of ATF6 in retinal angiogenesis, GRP78 expression is elevated in pathological neovascular lesions, and GRP78 has been shown to actively modulate inflammatory and angiogenic signaling through both intracellular and cell surface-associated mechanisms [58–60]. Cell surface GRP78 activates NFκB, ERK1/2, and AKT, which are known to regulate CXCL1/2 and IL-8 transcription in the modulation of VEGFA signaling in endothelial cells [61]. In retinal and vascular disease models, attenuation of ER stress signaling suppresses inflammatory chemokine expression and pathological angiogenesis [62, 63]. Together, these studies support a unified model in which OIR-induced activation of the ATF6-GRP78 signaling axis functions upstream of CXCL1/2 and IL-8 to mediate VEGFA-dependent pathological retinal neovascularization. However, currently it is unknown whether ATF6-GRP78 signaling axis plays a direct or permissive role in the induction of expression of these inflammatory chemokines and their receptor in the modulation of OIR-induced retinal neovascularization.

VEGFA binds preferentially to VEGFR2 whereas VEGFC and VEGFD bind to VEGFR3 in mediating angiogenesis and lymphangiogenesis, respectively [29, 30]. Besides, some studies have reported that both these receptors were involved in VEGFA-induced angiogenesis independently [64, 65]. On the other hand, some other studies have demonstrated that both the receptors interact with each other in modulating angiogenesis [29, 30]. Previously, we have shown that VEGFA induces VEGFR3 expression via VEGFR2-mediated PKCθ-JunB signaling axis in the regulation of pathological retinal angiogenesis [31]. We have also reported that VEGFR2-dependent VEGFR3 expression leads to activation of ATF6-GRP78 signaling in response to VEGFA/OIR in the retina [22]. In the present study, we show that silencing either VEGFR2 or VEGFR3 levels suppresses VEGFA-induced ATF6 activation and GRP78 induction leading to inhibition of CXCL1/2 and CXCR2 expression in MRMVECs/retina and IL-8 and CXCR2 expression in HRMVECs. In addition, VEGFR2 depletion abolished VEGFA-induced VEGFR3 expression, whereas VEGFR3 silencing had no effect on VEGFR2 levels, indicating a hierarchical signaling cascade in which VEGFR2-dependent VEGFR3 expression is required for activation of ATF6-GRP78 signaling. Collectively, these data support a model in which VEGFR2→VEGFR3→ATF6→GRP78→CXCL1/2 or IL-8 signaling leading to activation of CXCR2 is required for VEGFA/OIR-induced pathological retinal neovascularization. Based on the findings from other laboratories [53–57] and the present findings, it is likely CXCL1/2 or IL-8 signaling acts upstream as well as downstream to VEGFA in the modulation of retinal neovascularization. In view of these observations, it is likely that targeting IL-8 signaling may be more desirable therapeutic approach than anti-VEGF therapy alone in treating retinal neovascular diseases.

## Materials and methods

### Reagents

Recombinant human VEGF165a (Cat. No. 293-VE-010/CF), recombinant human IL-8 (Cat. No. MAB208-SP), recombinant mouse VEGF164a (Cat. No. 493-MV025/CF), recombinant mouse CXCL1 (Cat. No. 453-KC-010/CF), recombinant mouse CXCL2 (Cat. No. 452-M2-010/CF), rat IgG2A isotype control (Cat. No. MAB006), human IL-8 neutralizing antibody (Cat. No. MAB208-SP), mouse CXCL1 neutralizing antibody (Cat. No. MAB453-SP), mouse CXCL2 neutralizing antibody (Cat. No. MAB452-SP), mouse CXCR2 neutralizing antibody (Cat. No. MAB2164) and human CXCR2 neutralizing antibody (Cat. No. MAB331) were bought from R&D Systems (Minneapolis, MN), Growth factor-reduced Matrigel (Cat. No. 354230) was purchased from BD Biosciences (Bedford, MA). Normal mouse IgG (sc-2025), normal rabbit IgG (sc-2027), anti-VEGFR3 (sc-514825), anti-GRP78 (sc-13539), and anti-β-actin (sc-47778) antibodies were obtained from Santa Cruz Biotechnology (Santa Cruz, CA). Anti-VEGFR2 (Cat. No. 55B11) antibody was purchased from Cell Signaling Technology (Beverly, MA). Anti-CXCL1 (Cat. No. PA-5-86508), anti-CXCL2 (Cat. No. 701126), anti-CXCR1 (Cat. No. PA1-29466), anti-CXCR2 (Cat. No. PA5-100951) and anti-IL-8 (Cat. No. M801) antibodies were purchased from Thermo Fisher Scientific (Waltham, MA). Anti-ATF6 (Cat. No. ab122897) was purchased from Abcam (Cambridge, MA). Human VEGFR2 siRNA (Id: s535305), human VEGFR3 siRNA (Id: 145459), human ATF6 siRNA (Id: s223544), human GRP78 siRNA (Id: s6979), mouse ATF6 siRNA (Id: s105468), and mouse GRP78 siRNA (Id: s67083), and mouse VEGFR2 siRNA (Id: s68715), were obtained from Thermo Scientific (Chicago, IL). Mouse VEGFR3 siRNA (L-045433-00) was obtained from Dharmacon (Pittsburgh, PA). Endothelial basal medium-2 (EBM-2, Cat. No. CC-4176), and EGM-2 “Bullet Kit” (Cat. No. CC-4176) were purchased from Lonza (Walkersville, MD). DMEM (Cat. No. 10569-010) and Pen/Strep (Cat. No. 15070-063) were bought from Gibco (Waltham, MA). FBS (Cat. No. S11150) was purchased from Atlanta Biologicals (Atlanta, GA). Cytodex microcarrier beads (Cat. No. C3278) and VEGFR3 kinase inhibitor MAZ51 (Cat. No. 676492) were obtained from Sigma Aldrich (St Louis, MO). Fibrinogen (Cat. No. 820224) was bought from MP Biomedicals LCC (Solon, OH). Click-iT™ EdU Imaging Kit (Cat. No. C10337) and lipofectamine 3000 (Cat. No. L-3000-015) transfection reagents were purchased from Thermo Fisher Scientific (Waltham, MA). Isolectin B4-594 (I21413), cell tracker green (C7025), goat anti-rabbit IgG-AlexaFluor 488 (A11034), goat anti-rat IgG-AlexaFluor 568 (A11077), and Prolong Gold antifade reagent were bought from Molecular Probes (Eugene, OR).

### Animals

C57BL/6J (WT) (strain code 027) pregnant mice at E16 were bought from Charles River Laboratories (Wilmington, MA). The mouse strain used in this study was free of RD1 and RD8 mutations [66, 67] as revealed by genotyping using specific primers (Table 1). Mice were fed with Tekland irradiated LM-485 diet (Cat # 7912) and maintained at the University of Tennessee Health Science Center’s vivarium. All experiments involving animals were approved by the Institutional Animal Care and Use Committee (IACUC) of the University of Tennessee Health Science Center, Memphis, TN.

**Table 1:** List of primers

Gene	Forward primers 5′→3′	Reverse primers 5′→3′
qRT-PCR primers		
Mouse CXCL1	GTGAGGACATGTGTGGGAGG	CGAGACCAGGAGAAACAGGG
Mouse CXCL2	GGCGGTCAAAAAGTTTGCCT	CAGGTACGATCCAGGCTTCC
Mouse β-Actin	AGCCATGTACGTAGCCATCC	CTCTCAGCTGTGGTGGTGAA
Human IL-8	TCTGCAGCTCTGTGTGAAGG	TTCCTTGGGGTCCAGACAGA
Human β-Actin	AGCCATGTACGTTGCTAT	GATGTCCACGTCACACTTCA

Gene	Primers 5′→3′
PCR primers	
RD1 mutation	WT forward: TACCCACCCTTCCTAATTTTTCTCAGC
	Mut forward: GTAAACAGCAAGAGGCTTTATTGGGAAC
	Common reverse: TGACAATTACTCCTTTTCCCTCAGTCTG
RD8 mutation	WT forward: GTGAAGACAGCTACAGTTCTGATC
	Mut forward: GCCCCTGTTTGCATGGAGGAAACTTGGAAGACAGCTACAGTTCTTCTG
	Common reverse: GCCCCATTTGCACACTGATGAC

### Cell culture

Human retinal microvascular endothelial cells (HRMVECs; Cat. No. ACBRI 181) were obtained from Cell Systems (Kirkland, WA). Mouse retinal microvascular endothelial cells (MRMVEC’s) were isolated as described previously [22]. Both the HRMVECs and MRMVEC’s were cultured in endothelial basal medium (EBM) supplemented with EGM-2 “Bullet Kit” to make EGM-2 growth medium. Human fibroblasts (Cat. No. PCS-201-013) were purchased from the American Type Culture Collection (Manassas, VA) and maintained in DMEM supplemented with 10% FBS and 100 U/ml Penicillin//100 µg/ml Streptomycin. All cells were grown at 37°C in a humidified incubator with 95% air and 5% CO₂. Experiments were conducted on HRMVECs and MRMVECs between passages 5 and 9, synchronized by incubation in EBM for 24 h.

### Transfections

To prepare the transfection mixture, first 20 µl of P3000 reagent was added to 360 µl of Opti-MEM and 20 µl of 20 µM siRNA and incubated at room temperature for 5 min. Then 400 µl of lipofectamine 3000 reagent was added drop wisely to the above mixture and kept at room temperature for 30 min to yield 0.5 µM siRNA. This entire mixture was added to the cells in 100 mm dish containing 3.2 ml of EBM to achieve siRNA concentration to 100 pmoles/ml and incubated for 6 h at 37°C. After 6 h of transfection, the cells were returned to EGM and cultured for an additional 24 h. The cells were then synchronized overnight in EBM and used as needed.

### Cell migration

Endothelial cell migration was measured using a wound-healing assay as described previously [68]. HRMVECs and MRMVECs were seeded at a density of 3 × 10⁴ cells/ml into each chamber of Ibidi culture inserts and allowed to grow to confluence. Upon confluence, the inserts were gently removed using sterile forceps to create a uniform gap, and 1 ml of EBM containing 5 mM hydroxyurea was added. Cells were then treated with and without VEGFA (40 ng/ml) or CXCL1 (50 ng/ml) or CXCL2 (50 ng/ml) or IL-8 (100 ng/ml) alone or in the indicated combinations for 24 h at 37°C in a humidified CO₂ incubator. Wherever indicated, the neutralizing CXCL1 (1 µg/ml), CXCL2 (0.4 µg/ml), CXCR2 (3 µg/ml) or IL-8 (0.4 µg/ml) Abs were added, alone or in the indicated combinations 45 min prior the treatments. The images of the same fields were taken at 0 h and 24 h using a Nikon Eclipse TS100 microscope at 10X magnification. Images were captured with a charge-coupled device color camera and recorded using Apple iMovie software (https://support.apple.com/kb/dl13?locale=en_US). Cell migration was calculated as the percentage of wound closure using the formula: [(total area − cell-free area)/total area] × 100.

### Cell proliferation

Cell proliferation was measured using the Click-iT™ EdU Imaging Kit following the manufacturer's protocol. Briefly MRMVECs or HRMVECs were seeded onto sterile glass coverslips at 3 X 105 cells/coverslip in a 6-well plate and allowed to adhere overnight in EGM. Then, cells were quiesced in EBM for 12 h followed by treatment with and without VEGFA (40 ng/ml), or CXCL1 (50 ng/ml) or CXCL2 (50 ng/ml) or IL-8 (100 ng/ml), alone or in the indicated combinations for 4 h. Wherever appropriate, neutralizing Abs against CXCL1 (1 µg/ml), CXCL2 (0.4 µg/ml), CXCR2 (3 µg/ml) or IL-8 (0.4 µg/ml) were added 1 h before the agonist. Two hours before the end of the treatment period, 10 µM EdU was added to the culture medium and incubated at 37°C. Cells were subsequently washed and fixed with 4% paraformaldehyde for 15 min at room temperature and permeabilized with 0.5% Triton X-100 in PBS for 20 min. EdU incorporation into newly synthesized DNA was detected by incubating cells for 30 min with the Click-iT™ reaction cocktail containing Alexa Fluor™ 488 azide, CuSO₄, and reaction buffer additive, prepared freshly according to the kit instructions. Nuclei were counterstained with Hoechst 33342 (5 µg/ml) for 15 min. Coverslips were mounted onto the glass slides using antifade mounting medium, and the images were observed under a Zeiss inverted fluorescence microscope (Zeiss Axiovision Observer.z1). The fluorescent images were captured with a Zeiss AxioCam MRm camera using the microscope operating and image analysis software (ZEN 2.6 blue edison). At least five random fields per slide were captured, and the EdU-positive nuclei were quantified using ImageJ 1.49v software. The proliferation index was expressed as the percentage of EdU-positive cells relative to the total number of nuclei.

### Sprouting

Three-dimensional endothelial sprouting assay was used as described previously [69]. Briefly, both MRMVECs or HRMVECs were labelled with a fluorescent cell tracker (1 µM), trypsinized, pelleted, and incubated with Cytodex microcarrier beads (100 µl of 2000 beads/ml) for 6 h to allow cells to attach. Nonadherent cells were removed by washing with PBS, and the coated beads were embedded in fibrin gel (fibrinogen 2 mg/ml, aprotinin 0.15 U/ml and thrombin 0.625 U/ml). Human fibroblasts suspended in EGM were overlaid on the top of the fibrin gel at 2 x 104 cells/chamber. VEGFA (40 ng/ml) CXCL1 (50 ng/ml), CXCL2 (50 ng/ml), IL-8 (100 ng/ml), alone or in the indicated combinations were added and incubated at 37 °C. After 6 h of incubation, the medium was replaced with fresh EGM containing the respective cytokines and incubation continued for an additional 5 days. Endothelial sprouting was under a Zeiss inverted fluorescence microscope (Zeiss Axiovision Observer.z1, 10X/NA, 0.45). The fluorescent images were captured with a Zeiss AxioCam MRm camera using the microscope operating and image analysis software (ZEN 2.6 blue edison) and sprouting was quantified as the number of sprouts per bead.

### Tube formation

To evaluate tube formation, 24-well plates were coated with 300 μl per well of growth factor-reduced matrigel and incubated at 37°C for 30 min to allow the gel to solidify. MRMVECs or HRMVECs were then seeded onto the Matrigel at 2 × 10⁵ cells per well in EBM containing with and without VEGFA (40 ng/ml) CXCL1 (50 ng/ml), CXCL2 (50 ng/ml), IL-8 (100 ng/ml), alone or in the indicated combinations and incubated at 37°C for 6 h, at which time the tube-like structures were visualized under an inverted phase-contrast microscope (Nikon Eclipse TS100). Wherever appropriate, neutralizing Abs against CXCL1 (1 µg/ml), CXCL2 (0.4 µg/ml), CXCR2 (3 µg/ml) or IL-8 (0.4 µg/ml) were added 1 h before the agonist. The images were captured with a charge-coupled device color camera using Apple iMovie 7.1.4 software. Tube formation was quantified by measuring tube length and the number of tubes using the Angiogenesis Analyzer plugin in ImageJ version 1.53 software and expressed in micrometers.

### qRT-PCR

After appropriate treatments, total RNA was isolated from MRMVECs or HRMVECs using TRIzol reagent as per the manufacturer’s protocol. Reverse transcription was performed with a High-Capacity cDNA Reverse Transcription kit (Applied Biosystems, Foster City, CA). Quantitative reverse transcription-PCR was then performed on a 7300 Real-Time PCR system (Applied Biosystems) using SYBR Green Master Mix (Applied Biosystems) as per the manufacturer’s instructions. Beta actin was used as endogenous control. The PCR amplifications were carried out on a 7300 Real-Time PCR system operated with SDS version 1.4 program and Delta Rn analysis method (Applied Biosystems). All the primers used for qRT-PCR in this study were listed in Table 1.

### Western blotting

After appropriate treatments, MRMVECs or HRMVECs were lysed in RIPA buffer (PBS containing 1% Nonidet P40, 0.5% sodium deoxycholate, 0.1% SDS) containing protease and phosphatase inhibitor cocktails (PMSF 100 μg/ml, aprotinin 100 μg/ml, leupeptin 1 μg/ml and sodium orthovanadate 1mM) on ice for 30 min. Cell lysates were cleared by centrifugation at 12000 rpm for 10 min at 4°C. Protein concentrations were determined using micro BCA protein assay kit. Equal amounts of protein (40 µg) from control and each treatment were separated on 0.1% SDS and 10% or 12% polyacrylamide gels and transferred electrophoretically (at 95 volts) for 2 h onto nitrocellulose membranes. Membranes were blocked with 5% non-fat dry milk or 5% BSA in TBS containing 0.1% Tween-20 for 1 h at room temperature and incubated overnight at 4°C with the indicated primary antibody. After washing with TBST (5 times, 5 min/each), the membranes were incubated with HRP-conjugated secondary antibodies for 1 h at room temperature. The membranes were washed with TBST for 3 times, 5 min each and once with PBS for 5 min and developed in enhanced chemiluminescence (ECL) reagent. The antigen-antibody complexes were detected by exposing the membrane to X-ray film in AFP Mini-Med 90 X-ray film processor. The band intensities were quantified densitometric analysis using ImageJ version 1.53 software.

### OIR model

To study retinal neovascularization, we used oxygen-induced retinopathy (OIR) mouse model as described previously [32]. C57BL/6J mouse pups along with their nursing dams were exposed to hyperoxic environment (75% oxygen) from postnatal day 7 (P7) to P12 in a specialized oxygen-regulated chamber (Biospherix, Ltd). Oxygen levels were monitored and maintained using an O₂ controller system. Neutralizing Abs (1 µg in 0.5 µl/eye) were administered by intravitreal injections on P11 and P12. Intravitreal injections were given under isoflurane anesthesia using a glass micropipette connected to a 33-gauge Hamilton syringe into the vitreous cavity. On P12, the mice pups along with their dams were returned to room air (21% oxygen) to induce relative retinal hypoxia. Mice pups of the same age were kept at room air to serve as controls. Care was taken to avoid damaging the lens. All pups were sacrificed at postnatal P15 or P17 and the eyes were enucleated using a blunt end forceps. Retinas were dissected out for retinal extract preparation or eyes were fixed in 4% paraformaldehyde for 1 hour at room temperature. After fixation, retinas were isolated, stained with isolectin B4, flat mounts were prepared, mounted on glass slides and examined under a Zeiss inverted fluorescence microscope (Zeiss Axiovision Observer.z1). The fluorescent images were captured with a Zeiss AxioCam MRm camera using the microscope operating and image analysis software (ZEN 2.6 blue edison) Retinal neovascularization was quantified using Nikon NIS-Elements software (version AR31) according to a protocol described previously [22, 31, 70]. For quantification, the isolectin B4 flat mount images were analyzed using the above software by setting a fluorescence intensity threshold point of 50. Regions with fluorescence intensity above this threshold were automatically highlighted in a selected pseudo color (red). The software generated values for primary fluorescence intensity (total fluorescence of the flat mount), binary fluorescence intensity (fluorescence of the highlighted regions), and total retinal area were exported to an Excel file and used to calculate vascular density and the percentage of neovascularization by applying the following formulas: Vasculature = total retinal area/primary fluorescence intensity; Percentage neovascularization = primary fluorescence intensity/binary fluorescence intensity × 100.

### Immunofluorescence staining

Normoxic and hypoxic (post-OIR) eyes were enucleated, fixed in 4% paraformaldehyde, placed into OCT compound, and 8 mm thick sections were cut from the central part of the retina. The retinal sections were washed in PBS for 5 min at RT, blocked in 5% normal goat serum and incubated with anti-rabbit ki67 antibody in combination with anti-mouse CD31 antibody at 1:100 dilution overnight at 4°C. Then, the slides were washed with PBS and incubated with Alexa Fluor 568-conjugated goat anti-rabbit and Alexa Fluor 488-conjugated goat anti-mouse secondary antibodies for 1 h at RT. The sections were washed with PBS, counterstained with Hoechst, mounted with a cover slip using ProLong Gold antifade reagent and observed under a Zeiss inverted microscope (Axiovision Observer.z1; 40/NA 0.6 or 10/NA 0.45), and the fluorescence images were captured by a Zeiss AxioCam MRm camera using the microscope operating and image analysis software Zen 2.6 (blue edition).

### Filopodia-like protrusions

Retinal flat mounts from normoxic mice and 120-h post-OIR mice were stained with isolectin B4 and examined with a Zeiss inverted microscope (Axiovision Observer.z1; 40/NA 0.6) and fluorescence images were captured using Zeiss AxioCam MRm camera and the microscope operating and image analysis software Zen 2.6 (blue edition). The filopodia-like protrusions from ECs at the vascular front were counted using NIH ImageJ (version 1.53) and expressed as protrusions per 100-μm vessel length.

### Avascular area

The avascular area was determined according to a method described previously [22, 31, 70]. Briefly, the images of retinal flat mounts stained with isolectin B4 were opened in Photoshop version 23 (Adobe, San Jose, CA). Using the Polygonal Lasso tool and selecting the Add to Selection function, the outer boundary of the retinal flat mount was selected to obtain the total retinal area. Using the same tools, the center was selected toward the optical nerve that marks the vaso-obliterated area, which is highlighted in the white color. Once the total and vaso-obliterated areas were outlined, then using the Refresh key, the number of pixels in those areas was recorded. The percent avascular area was then calculated by dividing the pixels in the vaso-obliterated area by the pixels in the total area and multiplying by 100.

### Statistical analysis

All the in vitro experiments were performed three times. The normality of the data was evaluated using the Anderson-Darling Normality Test, while the F-test was used to assess the equality of group variance. Normally distributed data with similar variance were analyzed by student’s t-test for two-group comparisons, one-way/two-way ANOVA for multiple group comparisons with Tukey’s or Bonferroni’s post-hoc tests. The group size for mice was determined by priori power analysis, where α = 0.05, beta = 0.20 and power = 0.80. Because mouse pups from P7-P17 were used in the OIR experiments and the pups are sexually immature, sex differences were not expected to influence the observations. Therefore, OIR experiments were not performed in a gender-specific manner. All the statistical analyses were conducted utilizing GraphPad Prism version 8.00 software. A minimum of seven mice were included in the in vivo experiments. One mouse was considered as one biological unit and multiple measurements from the same experiment or animal were averaged to generate one value. The data are reported as Mean ± SD, and P < 0.05 was considered statistically significant.

## Supplementary Information

Below is the link to the electronic supplementary material.

Supplementary Material 1

## Data Availability

No datasets were generated or analysed during the current study.

## References

[1] Song H, Li Q, Gui X, Fang Z, Zhou W, Wang M, Jiang Y, Geng A, Shen X, Liu Y, Zhang H (2025) Endothelial protein C receptor promotes retinal neovascularization through heme catabolism. Nat Commun 16(1):1603. 10.1038/s41467-025-56810-0 PMC11825934 39948347

[2] Campochiaro PA (2013) Ocular neovascularization. J Mol Med 91(3):311–321. 10.1007/s00109-013-0993-5 PMC3584193 23329331

[3] Campochiaro PA (2015) Molecular pathogenesis of retinal and choroidal vascular diseases. Prog Retin Eye Res 49:67–81. 10.1016/j.preteyeres.2015.06.002 PMC4651818 26113211

[4] Friedlander M (2007) Fibrosis and diseases of the eye. J Clin Invest 117(3):576–586. 10.1172/JCI31030 PMC1804382 17332885

[5] Lazarus A, Keshet E (2011) Vascular endothelial growth factor and vascular homeostasis. Proc Am Thorac Soc 8(6):508–511. 10.1513/pats.201102-021MW 22052928

[6] Cao Y, Langer R, Ferrara N (2023) Targeting angiogenesis in oncology, ophthalmology and beyond. Nat Rev Drug Discov 22(6):476–495. 10.1038/s41573-023-00671-z 37041221

[7] Olson TS, Ley K (2002) Chemokines and chemokine receptors in leukocyte trafficking. Am J Physiol Regul Integr Comp Physiol 283(1):R7–R28. 10.1152/ajpregu.00738.200 12069927

[8] Strieter RM, Burdick MD, Gomperts BN, Belperio JA, Keane MP (2005) CXC chemokines in angiogenesis. Cytokine Growth Factor Rev 16(6):593–609. 10.1016/j.cytogfr.2005.04.007 16046180

[9] Romagnani P, Lasagni L, Annunziato F, Serio M, Romagnani S (2004) CXC chemokines: the regulatory link between inflammation and angiogenesis. Trends Immunol 25(4):201–209. 10.1016/j.it.2004.02.006 15039047

[10] Heidemann J, Ogawa H, Dwinell MB, Rafiee P, Maaser C, Gockel HR, Otterson MF, Ota DM, Lugering N, Domschke W, Binion DG (2003) Angiogenic effects of interleukin 8 (CXCL8) in human intestinal microvascular endothelial cells are mediated by CXCR2. J Biol Chem 278(10):8508–8515. 10.1074/jbc.M208231200 12496258

[11] Yu Y, Zhang J, Zhu R, Zhao R, Chen J, Jin J, Tian Y, Su SB (2017) The profile of angiogenic factors in vitreous humor of the patients with proliferative diabetic retinopathy. Curr Mol Med 17(4):280–286. 10.2174/1566524017666171106111440 29110608

[12] Mason RH, Minaker SA, Lahaie Luna G, Bapat P, Farahvash A, Garg A, Bhambra N, Muni RH (2022) Changes in aqueous and vitreous inflammatory cytokine levels in proliferative diabetic retinopathy: a systematic review and meta-analysis. Eye. 10.1038/s41433-022-02127-x 35672457

[13] Russo RC, Garcia CC, Teixeira MM, Amaral FA (2014) The CXCL8/IL-8 chemokine family and its receptors in inflammatory diseases. Expert Rev Clin Immunol 10(5):593–619. 10.1586/1744666X.2014.894886 24678812

[14] Zhou C, Gao Y, Ding P, Wu T, Ji G (2023) The role of CXCL family members in different diseases. Cell Death Discov 9(1):212. 10.1038/s41420-023-01524-9 PMC10314943 37393391

[15] Apte RS, Chen DS, Ferrara N (2019) VEGF in signaling and disease: beyond discovery and development. Cell 176(6):1248–1264. 10.1016/j.cell.2019.01.021 PMC6410740 30849371

[16] Ahmad A, Nawaz MI (2022) Molecular mechanism of VEGF and its role in pathological angiogenesis. J Cell Biochem 123(12):1938–1965. 10.1002/jcb.30344 36288574

[17] Pugh CW, Ratcliffe PJ (2003) Regulation of angiogenesis by hypoxia: role of the HIF system. Nat Med 9(6):677–684. 10.1038/nm0603-677 12778166

[18] Bartoszewska S, Collawn JF (2020) Unfolded protein response (UPR) integrated signaling networks determine cell fate during hypoxia. Cell Mol Biol Lett 25(1):18. 10.1186/s11658-020-00212-1 PMC7071609 32190062

[19] Lei Y, Yu H, Ding S, Liu H, Liu C, Fu R (2024) Molecular mechanism of ATF6 in unfolded protein response and its role in disease. Heliyon 10(5):e25937. 10.1016/j.heliyon.2024.e25937 PMC10907738 38434326

[20] Ghavami S, Aghaei M (2026) Pharmacological modulation of ATF6: exploiting a stress-integrative node to overcome drug resistance. Drug Discov Today Article ID. 10.1016/j.drudis.2026.104734. :104734 42425265

[21] Zhang SX, Ma JH, Bhatta M, Fliesler SJ, Wang JJ (2015) The unfolded protein response in retinal vascular diseases: implications and therapeutic potential beyond protein folding. Prog Retin Eye Res 45:111–131. 10.1016/j.preteyeres.2014.12.001 PMC4339403 25529848

[22] Kumar R, Rao GN (2024) Glucose-regulated protein 78, via releasing β-catenin from adherens junctions, facilitates its interaction with STAT3 in mediating retinal neovascularization. Am J Pathol 194(12):2356–2381. 10.1016/j.ajpath.2024.08.005 PMC11587869 39222910

[23] Aiello LP, Avery RL, Arrigg PG, Keyt BA, Jampel HD, Shah ST, Pasquale LR, Thieme H, Iwamoto MA, Park JE, Nguyen HV (1994) Vascular endothelial growth factor in ocular fluid of patients with diabetic retinopathy and other retinal disorders. N Engl J Med 331(22):1480–1487. 10.1056/NEJM199412013312203 7526212

[24] Pierce EA, Foley ED, Smith LE (1996) Regulation of vascular endothelial growth factor by oxygen in a model of retinopathy of prematurity. Arch Ophthalmol 114(10):1219–1228. 10.1001/archopht.1996.01100140419009 8859081

[25] Canataroglu H, Varinli I, Ozcan AA, Canataroglu A, Doran F, Varinli S (2005) Interleukin (IL)-6, interleukin (IL)-8 levels and cellular composition of the vitreous humor in proliferative diabetic retinopathy, proliferative vitreoretinopathy, and traumatic proliferative vitreoretinopathy. Ocul Immunol Inflamm 13(5):375–381. 10.1080/09273940490518900 16422002

[26] Wang Y, Gao S, Cao F, Yang H, Lei F, Hou S (2024) Ocular immune-related diseases: molecular mechanisms and therapy. MedComm 5(12):e70021. 10.1002/mco2.70021 PMC11604294 39611043

[27] Chappell JC, Wiley DM, Bautch VL (2011) Regulation of blood vessel sprouting. Semin Cell Dev Biol 22(9):1005–1011. 10.1016/j.semcdb.2011.10.006 PMC4521217 22020130

[28] Herbert SP, Stainier DY (2011) Molecular control of endothelial cell behaviour during blood vessel morphogenesis. Nat Rev Mol Cell Biol 12(9):551–564. 10.1038/nrm3176 PMC3319719 21860391

[29] Shaik F, Cuthbert GA, Homer-Vanniasinkam S, Muench SP, Ponnambalam S, Harrison MA (2020) Structural basis for vascular endothelial growth factor receptor activation and implications for disease therapy. Biomolecules. 10.3390/biom10121673 PMC7765180 33333800

[30] Schoofs H, Zhang Y, Ortsäter H, Lytvyn M, Benedito R, Mäkinen T (2026) VEGFR2 is required for VEGF-C–VEGFR3–PI3Kα-mediated sprouting lymphangiogenesis. Nat Commun 17(1):4380. 10.1038/s41467-026-73013-3 PMC13179370 42140900

[31] Kumar R, Mani AM, Singh NK, Rao GN (2020) PKCθ-JunB axis via upregulation of VEGFR3 expression mediates hypoxia-induced pathological retinal neovascularization. Cell Death Dis 11(5):325. 10.1038/s41419-020-2522-0 PMC7206019 32382040

[32] Smith LE, Wesolowski E, McLellan A, Kostyk SK, D’Amato R, Sullivan R, D’Amore PA (1994) Oxygen-induced retinopathy in the mouse. Invest Ophthalmol Vis Sci 35(1):101–111 PMID: 7507904 7507904

[33] Srejovic JV, Muric MD, Jakovljevic VL, Srejovic IM, Sreckovic SB, Petrovic NT, Todorovic DZ, Bolevich SB, Sarenac Vulovic TS (2024) Molecular and cellular mechanisms involved in the pathophysiology of retinal vascular disease—interplay between inflammation and oxidative stress. Int J Mol Sci 25(21):11850. 10.3390/ijms252111850 PMC11546760 39519401

[34] Miller JW (2016) Beyond VEGF-The Weisenfeld Lecture. Invest Ophthalmol Vis Sci 57(15):6911–6918. 10.1167/iovs.16-21201 PMC5214225 28027565

[35] Sharma D, Zachary I, Jia H (2023) Mechanisms of acquired resistance to anti-VEGF therapy for neovascular eye diseases. Invest Ophthalmol Vis Sci 64(5):28. 10.1167/iovs.64.5.28 PMC10230445 37252731

[36] Muniyandi A, Hartman GD, Song Y, Mijit M, Kelley MR, Corson TW (2023) Beyond VEGF: targeting inflammation and other pathways for treatment of retinal disease. J Pharmacol Exp Ther 386(1):15–25. 10.1124/jpet.122.001563 PMC10289243 37142441

[37] Goczalik I, Ulbricht E, Hollborn M, Raap M, Uhlmann S, Weick M, Pannicke T, Wiedemann P, Bringmann A, Reichenbach A, Francke M (2008) Expression of CXCL8, CXCR1, and CXCR2 in neurons and glial cells of the human and rabbit retina. Invest Ophthalmol Vis Sci 49(10):4578–4589. 10.1167/iovs.08-1887 18552386

[38] Monickaraj F, Acosta G, Cabrera AP, Das A (2023) Transcriptomic profiling reveals chemokine CXCL1 as a mediator for neutrophil recruitment associated with blood-retinal barrier alteration in diabetic retinopathy. Diabetes 72(6):781–794. 10.2337/db22-0619 PMC10202768 36930735

[39] Liyanage SE, Fantin A, Villacampa P, Lange CA, Denti L, Cristante E, Smith AJ, Ali RR, Luhmann UF, Bainbridge JW, Ruhrberg C (2016) Myeloid-derived vascular endothelial growth factor and hypoxia-inducible factor are dispensable for ocular neovascularization—brief report. Arterioscler Thromb Vasc Biol 36(1):19–24. 10.1161/ATVBAHA.115.30668 PMC4684248 26603154

[40] Wang S, Bai J, Zhang YL, Lin QY, Han X, Qu WK, Zhang PF, Ge YS, Zhao Q, Li HH (2022) CXCL1-CXCR2 signalling mediates hypertensive retinopathy by inducing macrophage infiltration. Redox Biol 56:102438. 10.1016/j.redox.2022.102438 PMC9418605 35981418

[41] Ren B, Li H, Liu S, Huang Z, Bai J, Jiang Z, Xu T, Gu B, Yu W, Sun L, Tan P (2025) Hic-5 deficiency attenuates MAFLD by inhibiting neutrophils migration via the CXCL1-CXCR2 axis. J Gastroenterol 60(12):1535–1553. 10.1007/s00535-025-02293-1 40924147

[42] Liu Y, Cox SR, Morita T, Kourembanas S (1995) Hypoxia regulates vascular endothelial growth factor gene expression in endothelial cells: identification of a 5′ enhancer. Circ Res 77(3):638–643. 10.1161/01.RES.77.3.638 7641334

[43] Forsythe JA, Jiang BH, Iyer NV, Agani F, Leung SW, Koos RD, Semenza GL (1996) Activation of vascular endothelial growth factor gene transcription by hypoxia-inducible factor 1. Mol Cell Biol 16(9):4604–4613. 10.1128/MCB.16.9.4604 PMC231459 8756616

[44] Petrovic MG, Korosec P, Kosnik M, Hawlina M (2007) Vitreous levels of interleukin-8 in patients with proliferative diabetic retinopathy. Am J Ophthalmol 143(1):175–176. 10.1016/j.ajo.2006.07.032 17188064

[45] Yu H, Huang X, Ma Y, Gao M, Wang O, Gao T, Shen Y, Liu X (2013) Interleukin-8 regulates endothelial permeability by down-regulation of tight junction but not dependent on integrins induced focal adhesions. Int J Biol Sci 9(9):966–979. 10.7150/ijbs.6996 PMC3805902 24155670

[46] Garcia MJ, Beall AL, Morales MS, Beatty NJ, Palmer SA, Jhala M, Drmanovic A, Priest S, Zhang Y, Yang R, Arellano K (2026) IL-8 receptor signaling as a novel target for angiogenic retinopathies. Angiogenesis 29(1):1–20. 10.1007/s10456-025-10015-7 PMC12579697 41176546

[47] Qian HY, Wei XH, Huang JO (2025) Inflammatory mechanisms in diabetic retinopathy: pathogenic roles and therapeutic perspectives. Am J Transl Res 17(8):6262–6274. 10.62347/GBFO5856 PMC12432744 40950276

[48] Ye J, Rawson RB, Komuro R, Chen X, Davé UP, Prywes R, Brown MS, Goldstein JL (2000) ER stress induces cleavage of membrane-bound ATF6 by the same proteases that process SREBPs. Mol Cell 6(6):1355–1364. 10.1016/S1097-2765(00)00133-7 11163209

[49] Shen J, Chen X, Hendershot L, Prywes R (2002) ER stress regulation of ATF6 localization by dissociation of BiP/GRP78 binding and unmasking of Golgi localization signals. Dev Cell 3(1):99–111. 10.1016/S1534-5807(02)00203-4 12110171

[50] Koumenis C, Naczki C, Koritzinsky M, Rastani S, Diehl A, Sonenberg N, Koromilas A, Wouters BG (2002) Regulation of protein synthesis by hypoxia via activation of the endoplasmic reticulum kinase PERK and phosphorylation of the translation initiation factor eIF2α. Mol Cell Biol 22(21):7405–7416. 10.1128/MCB.22.21.7405-7416.2002 PMC135664 12370288

[51] Wu J, Rutkowski DT, Dubois M, Swathirajan J, Saunders T, Wang J, Song B, Yau GD, Kaufman RJ (2007) ATF6α optimizes long-term endoplasmic reticulum function to protect cells from chronic stress. Dev Cell 13(3):351–364. 10.1016/j.devcel.2007.07.005 17765679

[52] Zhang K, Kaufman RJ (2008) From endoplasmic-reticulum stress to the inflammatory response. Nature 454(7203):455–462. 10.1038/nature07203 PMC2727659 18650916

[53] Yamazaki H, Hiramatsu N, Hayakawa K, Tagawa Y, Okamura M, Ogata R, Huang T, Nakajima S, Yao J, Paton AW, Paton JC (2009) Activation of the Akt-NF-κB pathway by subtilase cytotoxin through the ATF6 branch of the unfolded protein response. J Immunol 183(2):1480–1487. 10.4049/jimmunol.0900017 PMC2762936 19561103

[54] Garg AD, Kaczmarek A, Krysko O, Vandenabeele P, Krysko DV, Agostinis P (2012) ER stress-induced inflammation: does it aid or impede disease progression? Trends Mol Med 18(10):589–598. 10.1016/j.molmed.2012.06.010 22883813

[55] Hoffmann A, Levchenko A, Scott ML, Baltimore D (2002) The IκB-NF-κB signaling module: temporal control and selective gene activation. Science 298(5596):1241–1245. 10.1126/science.1071914 12424381

[56] Addison CL, Daniel TO, Burdick MD, Liu H, Ehlert JE, Xue YY, Buechi L, Walz A, Richmond A, Strieter RM (2000) The CXC chemokine receptor 2, CXCR2, is the putative receptor for ELR + CXC chemokine-induced angiogenic activity. J Immunol 65(9):5269–5277. 10.4049/jimmunol.165.9.5269 11046061

[57] Waugh DJ, Wilson C (2008) The interleukin-8 pathway in cancer. Clin Cancer Res 14(21):6735–6741. 10.1158/1078-0432.CCR-07-4843 18980965

[58] Dong D, Ni M, Li J, Xiong S, Ye W, Virrey JJ, Mao C, Ye R, Wang M, Pen L, Dubeau L (2008) Critical role of the stress chaperone GRP78/BiP in tumor proliferation, survival, and tumor angiogenesis in transgene-induced mammary tumor development. Cancer Res 68(2):498–505. 10.1158/0008-5472.CAN-07-2950 18199545

[59] Lee AS (2014) Glucose-regulated proteins in cancer: molecular mechanisms and therapeutic potential. Nat Rev Cancer 14(4):263–276. 10.1038/nrc3701 PMC4158750 24658275

[60] Luo B, Lee AS (2013) The critical roles of endoplasmic reticulum chaperones and unfolded protein response in tumorigenesis and anticancer therapies. Oncogene 32(7):805–818. 10.1038/onc.2012.130 PMC3819728 22508478

[61] Misra UK, Deedwania R, Pizzo SV (2006) Activation and cross-talk between Akt, NF-kappaB, and unfolded protein response signaling in 1-LN prostate cancer cells consequent to ligation of cell surface-associated GRP78. J Biol Chem 281(19):13694–13707. 10.1074/jbc.M511694200 16543232

[62] Li J, Wang JJ, Yu Q, Wang M, Zhang SX (2009) Endoplasmic reticulum stress is implicated in retinal inflammation and diabetic retinopathy. FEBS Lett 583(9):1521–1527. 10.1016/j.febslet.2009.04.007 PMC2691649 19364508

[63] Zhang SX, Sanders E, Wang JJ (2011) Endoplasmic reticulum stress and inflammation: mechanisms and implications in diabetic retinopathy. J Ocul Biol Dis Infor 4(1–2):51–61. 10.1007/s12177-011-9075-5 PMC3342410 23330021

[64] Zarkada G, Heinolainen K, Makinen T, Kubota Y, Alitalo K (2015) VEGFR3 does not sustain retinal angiogenesis without VEGFR2. Proc Natl Acad Sci USA 112(3):761–766. 10.1073/pnas.1423278112 PMC4311859 25561555

[65] Dumont DJ, Jussila L, Taipale J, Lymboussaki A, Mustonen T, Pajusola K, Breitman M, Alitalo K (1998) Cardiovascular failure in mouse embryos deficient in VEGF receptor-3. Science 282(5390):946–949. 10.1126/science.282.5390.946 9794766

[66] Mattapallil MJ, Wawrousek EF, Chan CC, Zhao H, Roychoudhury J, Ferguson TA, Caspi RR (2012) The Rd8 mutation of the Crb1 gene is present in vendor lines of C57BL/6 N mice and embryonic stem cells and confounds ocular induced mutant phenotypes. Invest Ophthalmol Vis Sci 53(6):2921–2927. 10.1167/iovs.12-9662 PMC3376073 22447858

[67] Collin GB, Won J, Hicks WL, Cook SA, Nishina PM, Naggert JK (2012) Meckelin is necessary for photoreceptor intraciliary transport and outer segment morphogenesis. Invest Ophthalmol Vis Sci 53(2):967–974. 10.1167/iovs.11-8766 PMC3317434 22247471

[68] Luo S, Mao C, Lee B, Lee AS (2006) GRP78/BiP is required for cell proliferation and protecting the inner cell mass from apoptosis during early mouse embryonic development. Mol Cell Biol 26(15):5688–5697. 10.1128/MCB.00779-06 PMC1592753 16847323

[69] Nakatsu MN, Hughes CC (2008) An optimized three-dimensional in vitro model for the analysis of angiogenesis. Methods Enzymol 443:65–82. 10.1016/S0076-6879(08)02004-1 18772011

[70] Connor KM, Krah NM, Dennison RJ, Aderman CM, Chen J, Guerin KI, Sapieha P, Stahl A, Willett KL, Smith LE (2009) Quantification of oxygen-induced retinopathy in the mouse: a model of vessel loss, vessel regrowth and pathological angiogenesis. Nat Protoc 2009 4(11):1565–1573. 10.1038/nprot.2009.187 PMC3731997 19816419

