# Supplementary material for: VEGFR2-dependent VEGFR3 expression leads to CXCL1/2 and CXCR2 induction in the development of retinal neovascularization
Source: Angiogenesis. 2026 Oct 25;29(4):82. doi: 10.1007/s10456-026-10104-1 (PMC13696613; doi:10.1007/s10456-026-10104-1)

Figure 1

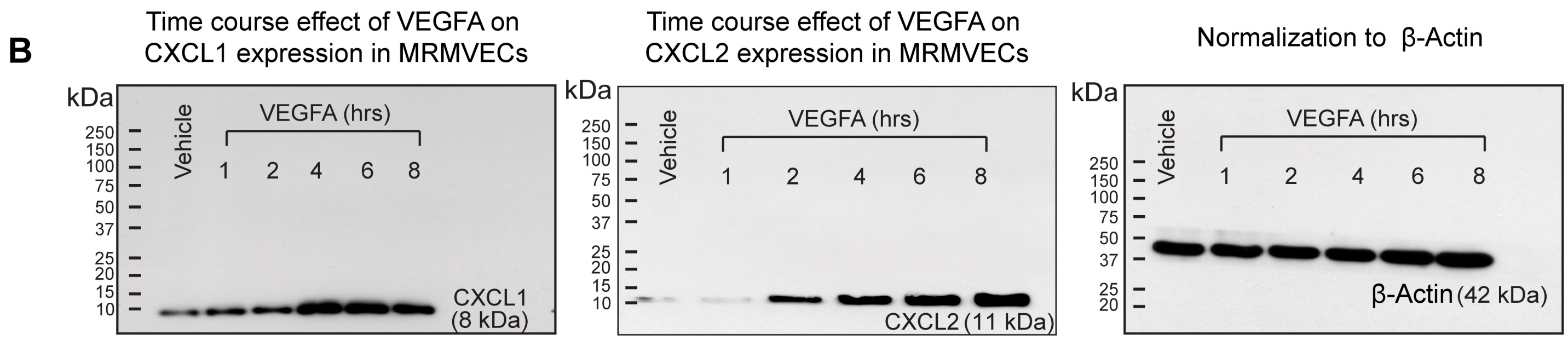

Figure 4

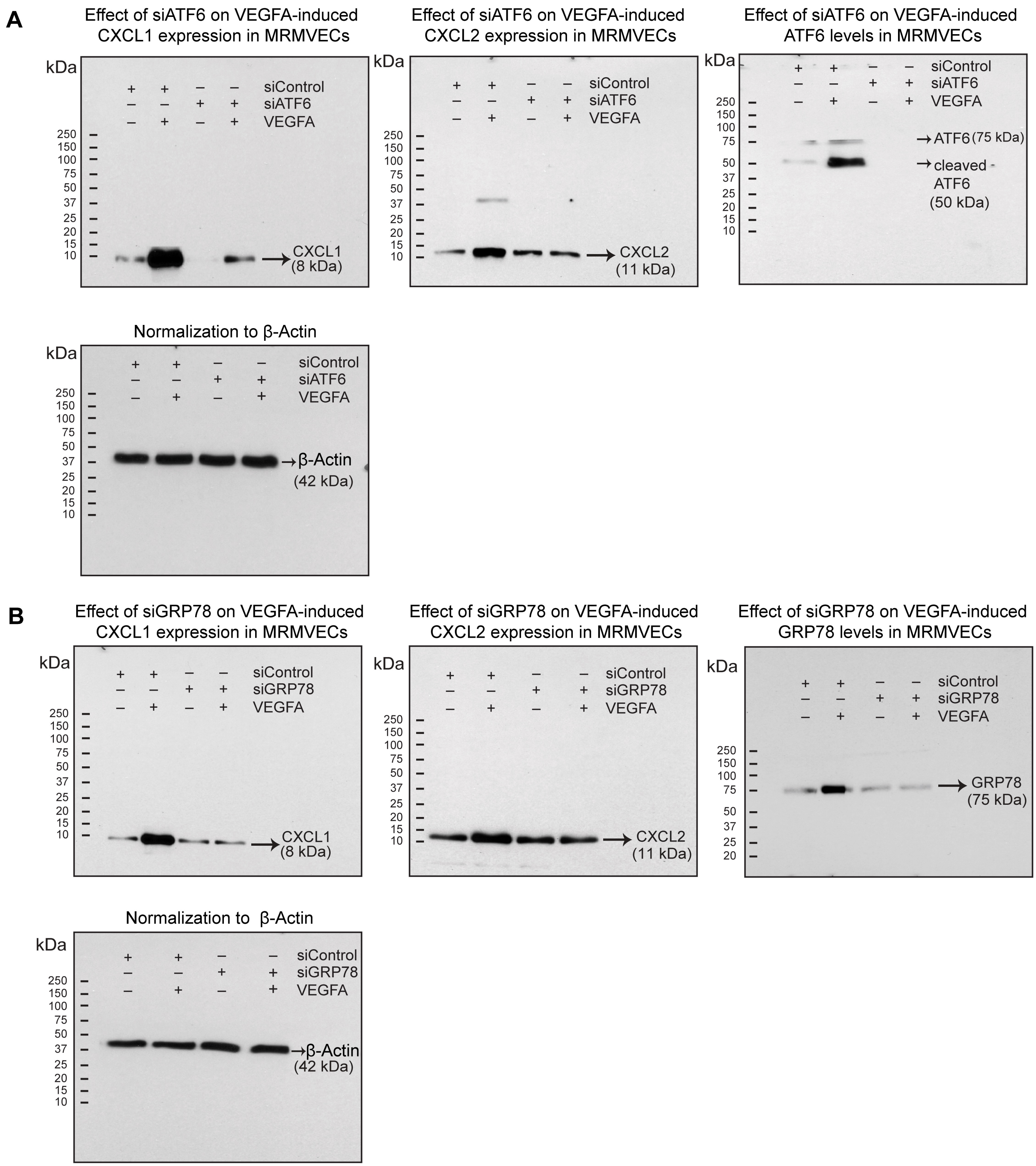

Figure 4

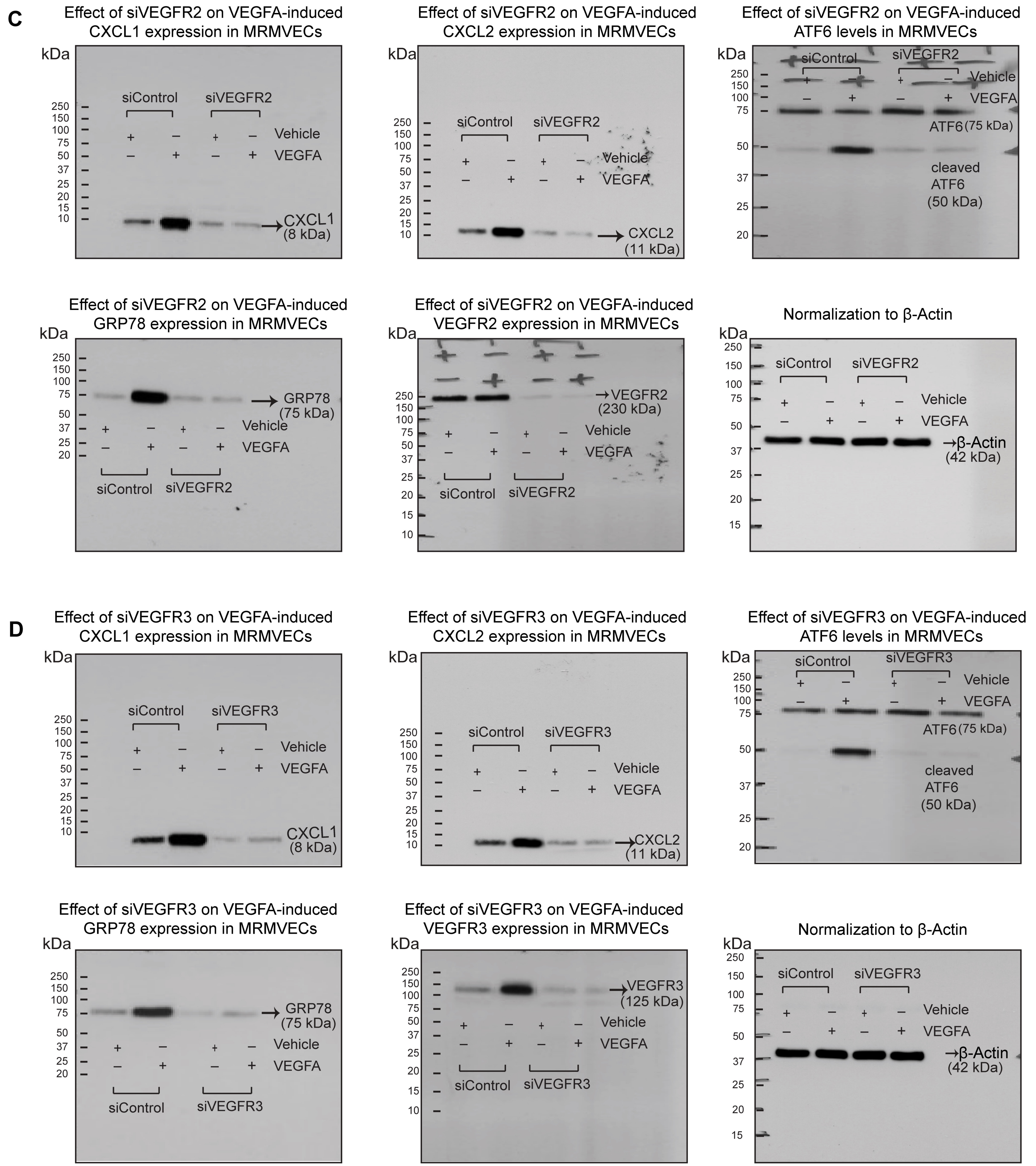
C
Effect of siVEGFR2 on VEGFA-induced CXCL1 expression in MRMVECs
kDa
250 150 100 75 50 37 25 20 15 10
siControl siVEGFR2
+ – + – Vehicle
– + – + VEGFA
CXCL1 (8 kDa)
Effect of siVEGFR2 on VEGFA-induced CXCL2 expression in MRMVECs
kDa
250 150 100 75 50 37 25 20 15 10
siControl siVEGFR2
+ – + – Vehicle
– + – + VEGFA
CXCL2 (11 kDa)
Effect of siVEGFR2 on VEGFA-induced ATF6 levels in MRMVECs
kDa
250 150 100 75 50 37 25 20
siControl siVEGFR2
+ – + – Vehicle
– + – + VEGFA
ATF6 (75 kDa)
cleaved ATF6 (50 kDa)
Effect of siVEGFR2 on VEGFA-induced GRP78 expression in MRMVECs
kDa
250 150 100 75 50 37 25 20
GRP78 (75 kDa)
+ – + – Vehicle
– + – + VEGFA
siControl siVEGFR2
Effect of siVEGFR2 on VEGFA-induced VEGFR2 expression in MRMVECs
kDa
250 150 100 75 50 37 25 20 15 10
VEGFR2 (230 kDa)
+ – + – Vehicle
– + – + VEGFA
siControl siVEGFR2
Normalization to β-Actin
kDa
250 150 100 75 50 37 25 20 15
siControl siVEGFR2
+ – + – Vehicle
– + – + VEGFA
β-Actin (42 kDa)
D
Effect of siVEGFR3 on VEGFA-induced CXCL1 expression in MRMVECs
kDa
250 150 100 75 50 37 25 20 15 10
siControl siVEGFR3
+ – + – Vehicle
– + – + VEGFA
CXCL1 (8 kDa)
Effect of siVEGFR3 on VEGFA-induced CXCL2 expression in MRMVECs
kDa
250 150 100 75 50 37 25 20 15 10
siControl siVEGFR3
+ – + – Vehicle
– + – + VEGFA
CXCL2 (11 kDa)
Effect of siVEGFR3 on VEGFA-induced ATF6 levels in MRMVECs
kDa
250 150 100 75 50 37 25 20
siControl siVEGFR3
+ – + – Vehicle
– + – + VEGFA
ATF6 (75 kDa)
cleaved ATF6 (50 kDa)
Effect of siVEGFR3 on VEGFA-induced GRP78 expression in MRMVECs
kDa
250 150 100 75 50 37 25 20
GRP78 (75 kDa)
+ – + – Vehicle
– + – + VEGFA
siControl siVEGFR3
Effect of siVEGFR3 on VEGFA-induced VEGFR3 expression in MRMVECs
kDa
250 150 100 75 50 37 25 20 15 10
VEGFR3 (125 kDa)
+ – + – Vehicle
– + – + VEGFA
siControl siVEGFR3
Normalization to β-Actin
kDa
250 150 100 75 50 37 25 20 15
siControl siVEGFR3
+ – + – Vehicle
– + – + VEGFA
β-Actin (42 kDa)

Figure 4

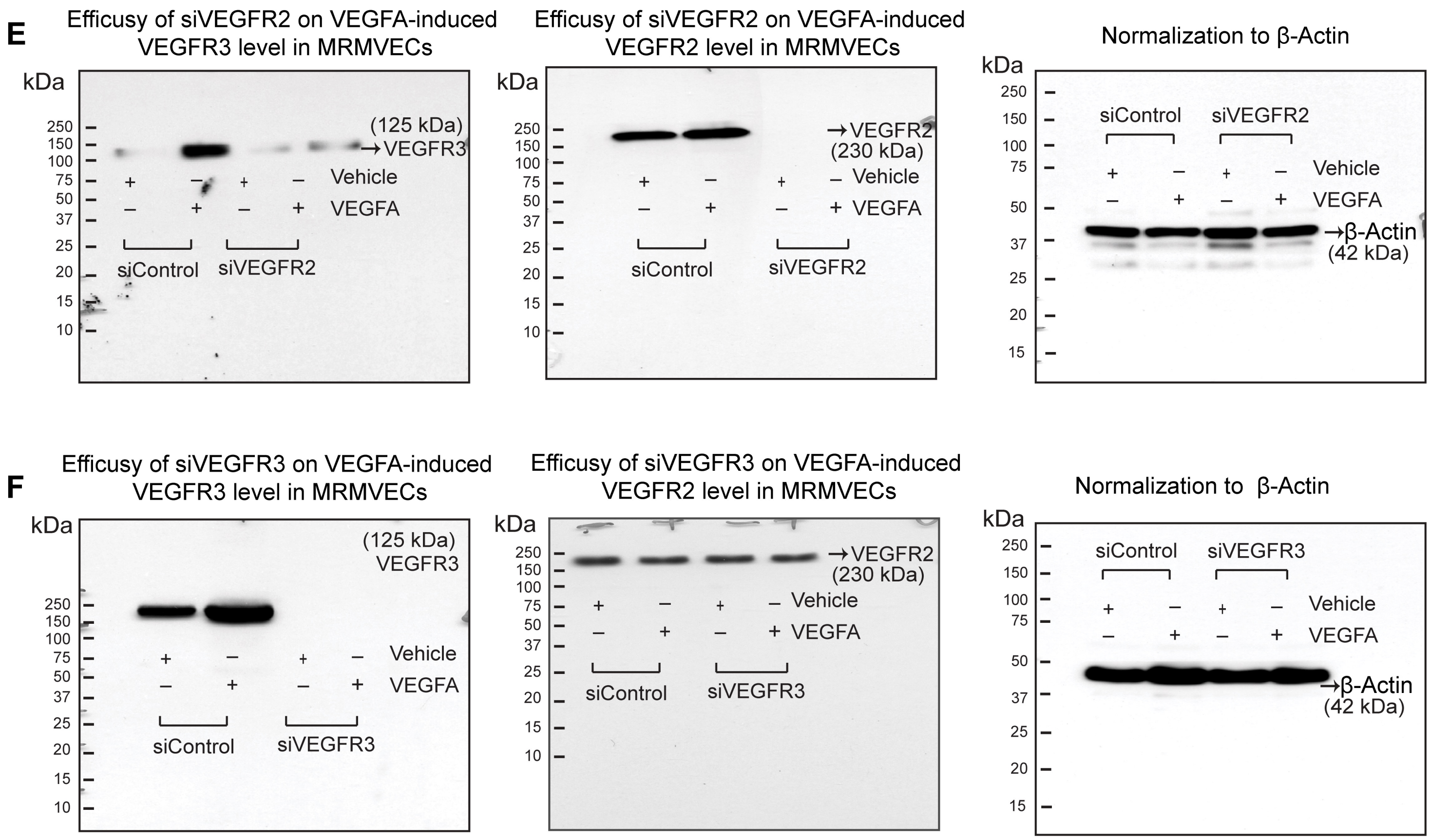

Figure 5

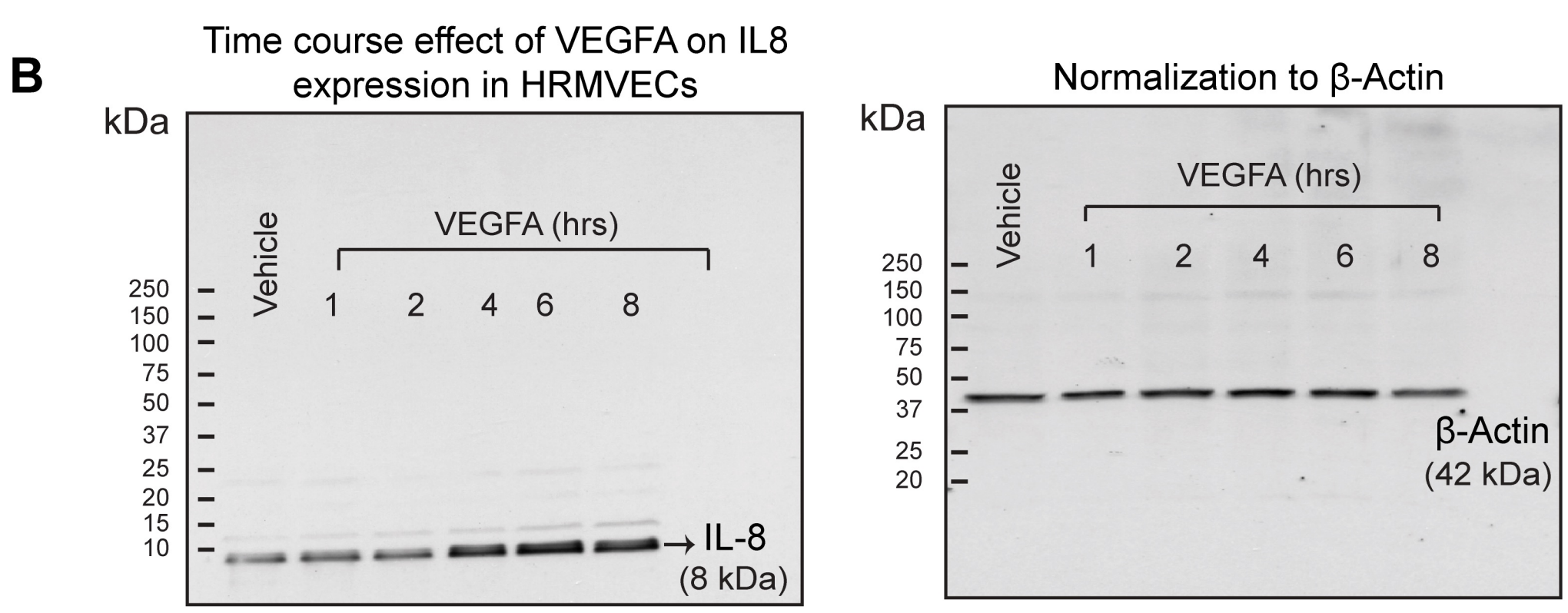

Figure 7

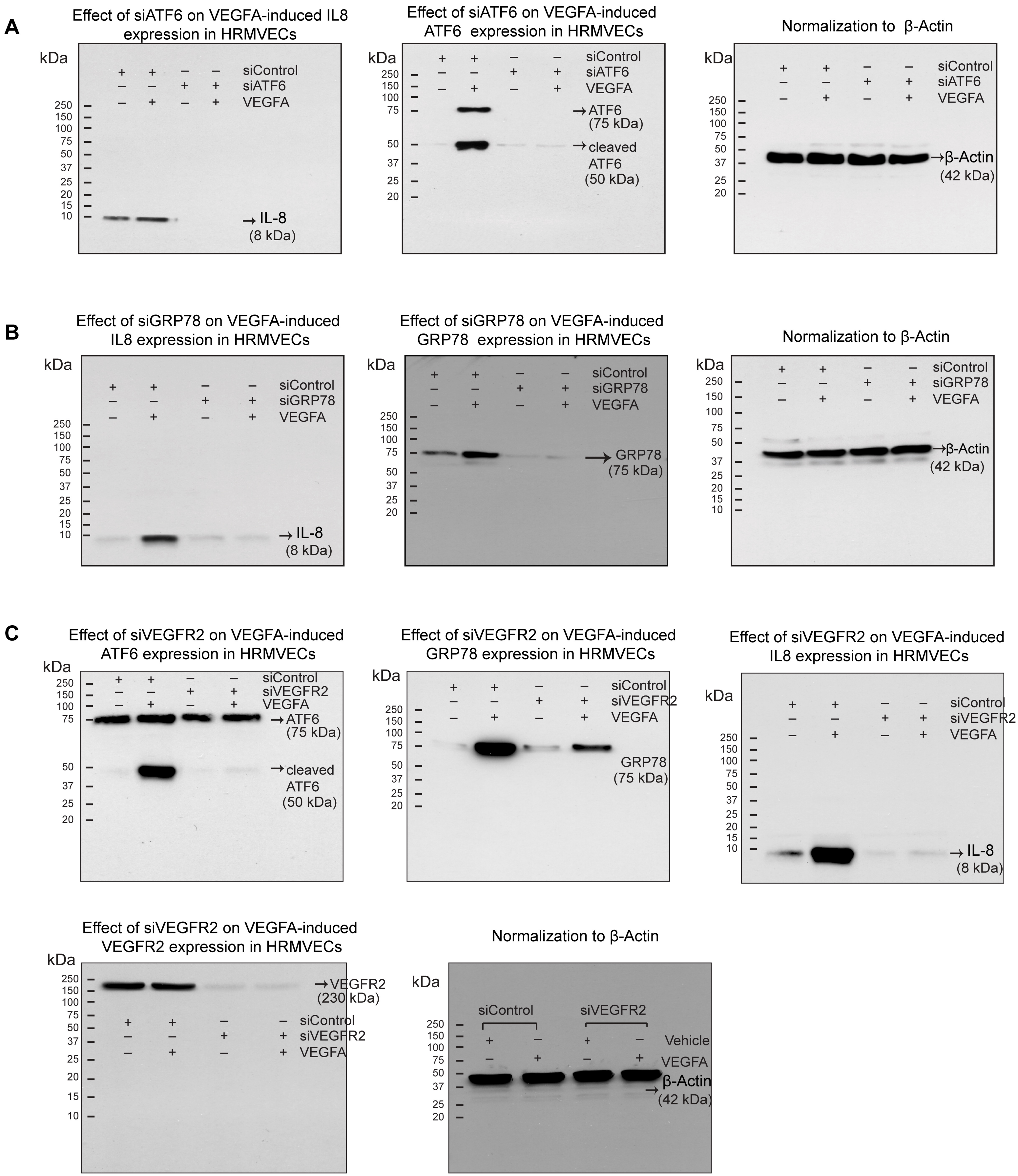
A
Effect of siATF6 on VEGFA-induced IL8 expression in HRMVECs
kDa
siControl
siATF6
VEGFA
250
150
100
75
50
37
25
20
15
10
IL-8
(8 kDa)
Effect of siATF6 on VEGFA-induced ATF6 expression in HRMVECs
ATF6
(75 kDa)
cleaved ATF6
(50 kDa)
Normalization to β-Actin
β-Actin
(42 kDa)
B
Effect of siGRP78 on VEGFA-induced IL8 expression in HRMVECs
siGRP78
Effect of siGRP78 on VEGFA-induced GRP78 expression in HRMVECs
GRP78
(75 kDa)
Normalization to β-Actin
C
Effect of siVEGFR2 on VEGFA-induced ATF6 expression in HRMVECs
siVEGFR2
Effect of siVEGFR2 on VEGFA-induced GRP78 expression in HRMVECs
Effect of siVEGFR2 on VEGFA-induced IL8 expression in HRMVECs
Effect of siVEGFR2 on VEGFA-induced VEGFR2 expression in HRMVECs
VEGFR2
(230 kDa)
Normalization to β-Actin
siControl
siVEGFR2
Vehicle
VEGFA

Figure 7

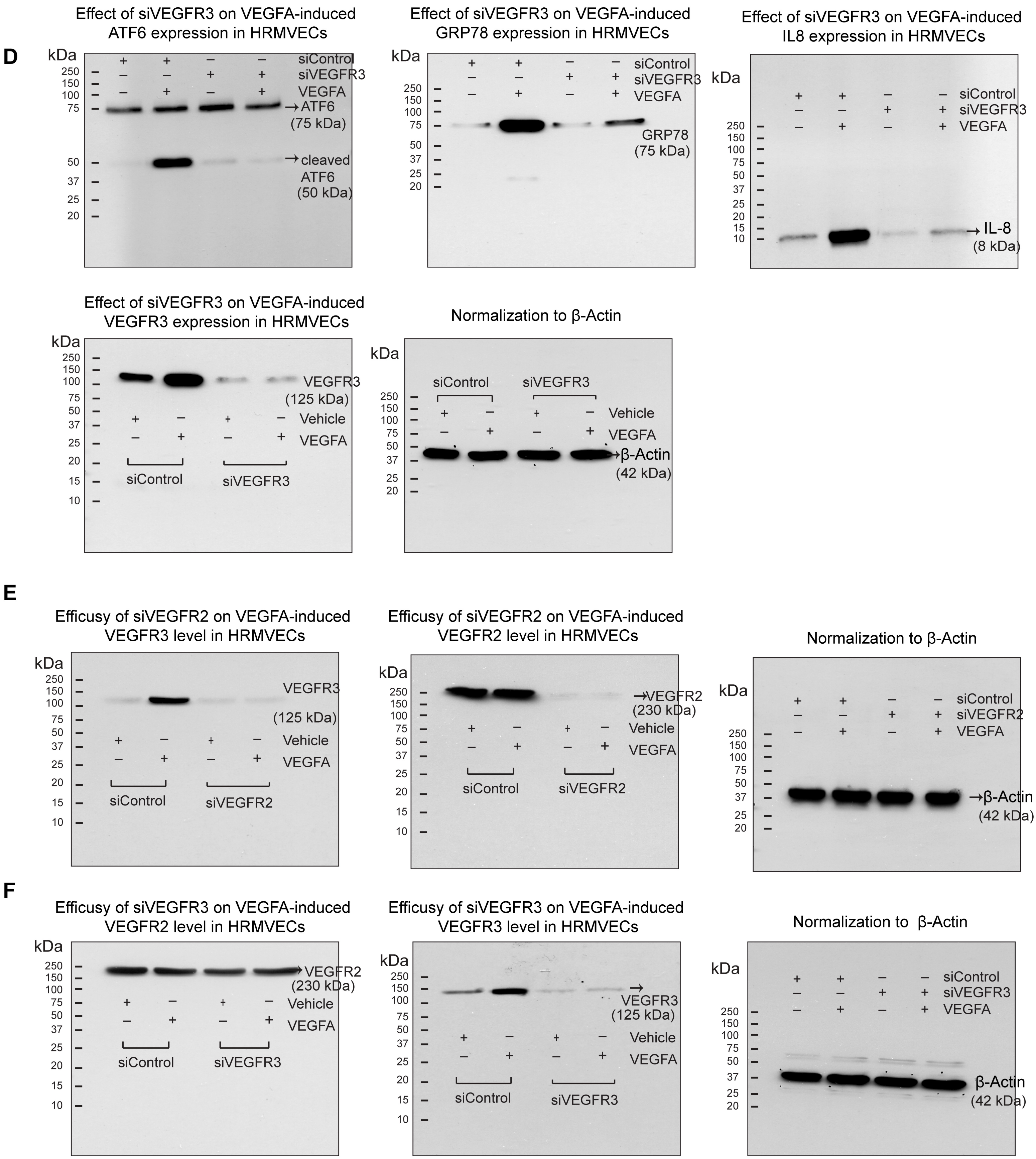
D
Effect of siVEGFR3 on VEGFA-induced ATF6 expression in HRMVECs
kDa
250
150
100
75
50
37
25
20
siControl
siVEGFR3
VEGFA
→ATF6 (75 kDa)
→cleaved ATF6 (50 kDa)
Effect of siVEGFR3 on VEGFA-induced GRP78 expression in HRMVECs
GRP78 (75 kDa)
Effect of siVEGFR3 on VEGFA-induced IL8 expression in HRMVECs
15
10
→IL-8 (8 kDa)
Effect of siVEGFR3 on VEGFA-induced VEGFR3 expression in HRMVECs
VEGFR3 (125 kDa)
Vehicle
VEGFA
siControl
siVEGFR3
Normalization to β-Actin
→β-Actin (42 kDa)
E
Efficusy of siVEGFR2 on VEGFA-induced VEGFR3 level in HRMVECs
VEGFR3 (125 kDa)
siVEGFR2
Efficusy of siVEGFR2 on VEGFA-induced VEGFR2 level in HRMVECs
→VEGFR2 (230 kDa)
Normalization to β-Actin
→β-Actin (42 kDa)
F
Efficusy of siVEGFR3 on VEGFA-induced VEGFR2 level in HRMVECs
→VEGFR2 (230 kDa)
Efficusy of siVEGFR3 on VEGFA-induced VEGFR3 level in HRMVECs
→ VEGFR3 (125 kDa)
Normalization to β-Actin
β-Actin (42 kDa)

Figure 8

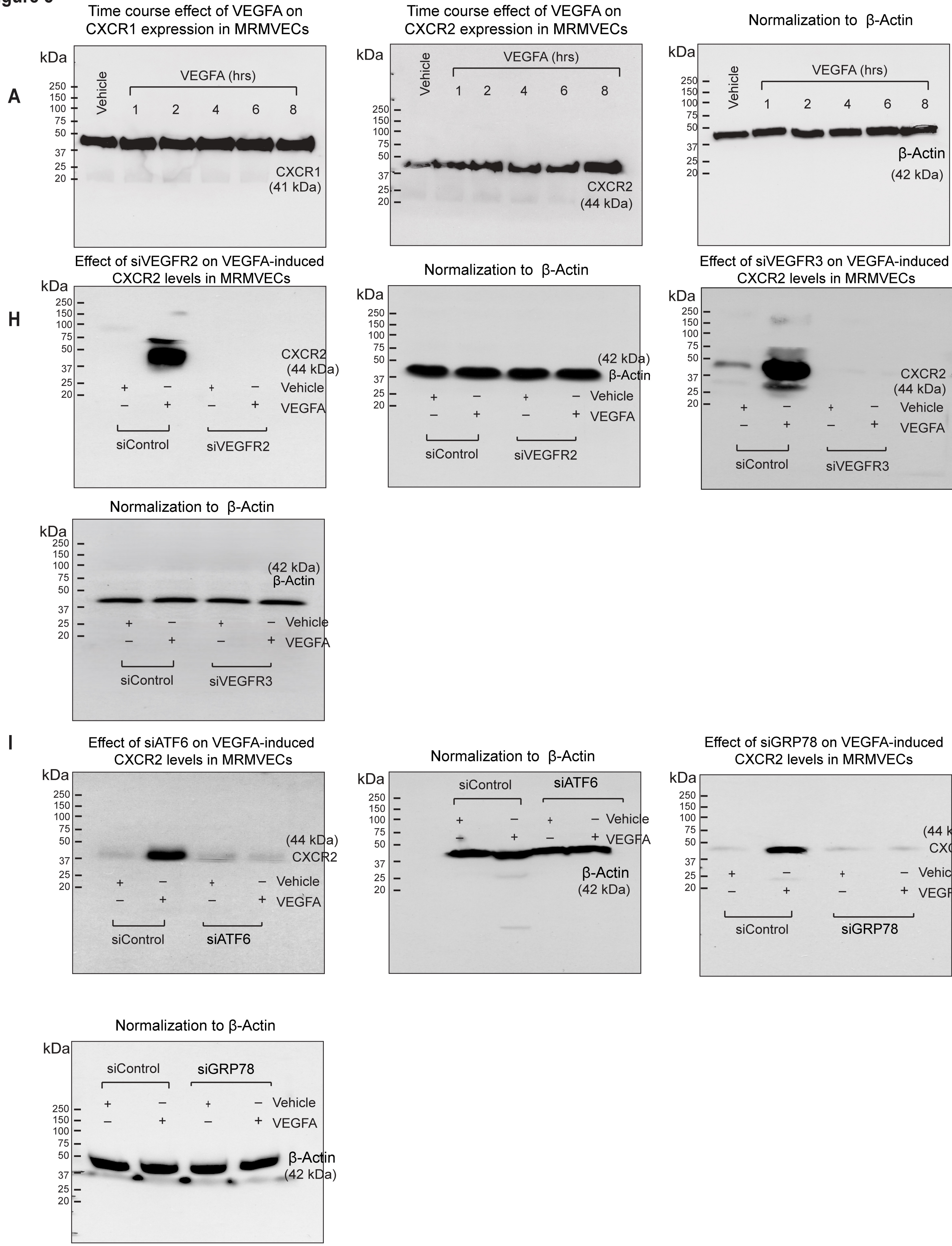

A
Time course effect of VEGFA on CXCR1 expression in MRMVECs
kDa
250
150
100
75
50
37
25
20
Vehicle
VEGFA (hrs)
1 2 4 6 8
CXCR1
(41 kDa)
Time course effect of VEGFA on CXCR2 expression in MRMVECs
VEGFA (hrs)
CXCR2
(44 kDa)
Normalization to β-Actin
β-Actin
(42 kDa)
H
Effect of siVEGFR2 on VEGFA-induced CXCR2 levels in MRMVECs
Vehicle
VEGFA
siControl
siVEGFR2
Effect of siVEGFR3 on VEGFA-induced CXCR2 levels in MRMVECs
siVEGFR3
I
Effect of siATF6 on VEGFA-induced CXCR2 levels in MRMVECs
siATF6
Effect of siGRP78 on VEGFA-induced CXCR2 levels in MRMVECs
siGRP78

Figure 9

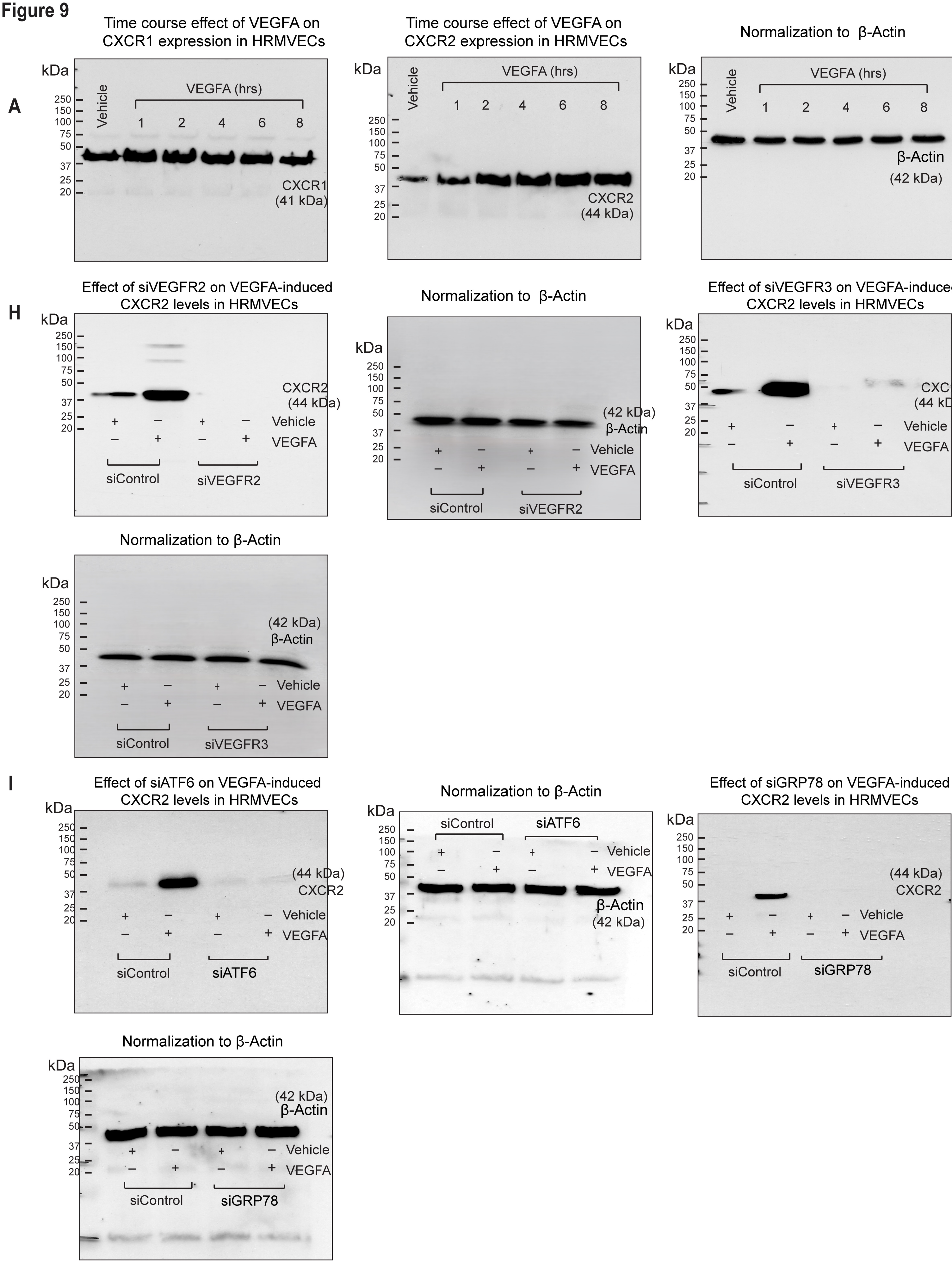
A
Time course effect of VEGFA on CXCR1 expression in HRMVECs
kDa
250
150
100
75
50
37
25
20
Vehicle
VEGFA (hrs)
1 2 4 6 8
CXCR1 (41 kDa)
Time course effect of VEGFA on CXCR2 expression in HRMVECs
CXCR2 (44 kDa)
Normalization to β-Actin
β-Actin (42 kDa)
H
Effect of siVEGFR2 on VEGFA-induced CXCR2 levels in HRMVECs
+ − + − Vehicle
− + − + VEGFA
siControl
siVEGFR2
Effect of siVEGFR3 on VEGFA-induced CXCR2 levels in HRMVECs
siVEGFR3
I
Effect of siATF6 on VEGFA-induced CXCR2 levels in HRMVECs
siATF6
Effect of siGRP78 on VEGFA-induced CXCR2 levels in HRMVECs
siGRP78

Figure 10A (left panel)

Effect of siATF6 on OIR-induced CXCL1 expression in mice pups

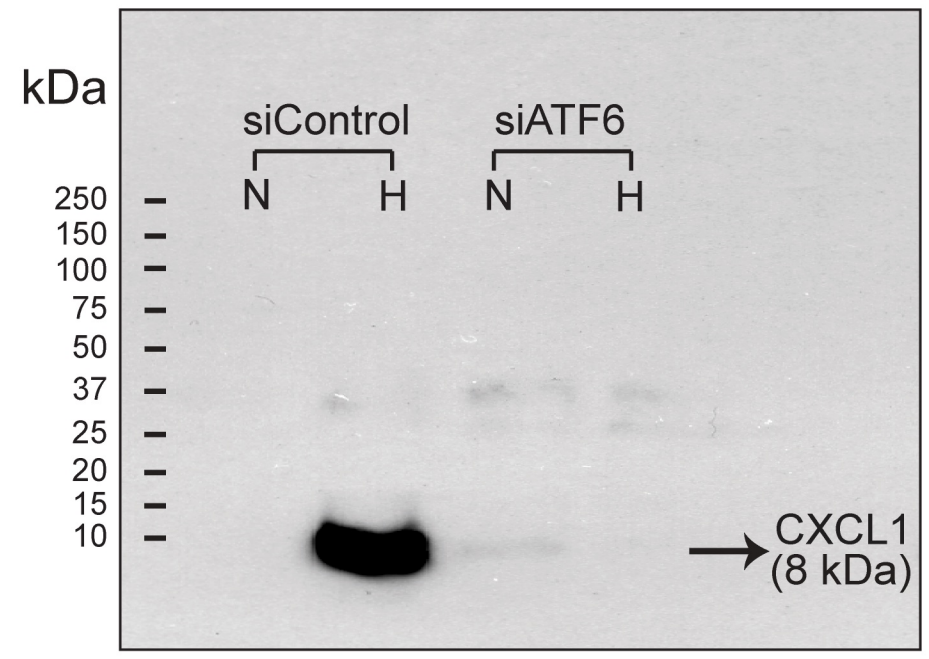

Effect of siATF6 on OIR-induced CXCL2 expression in mice pups

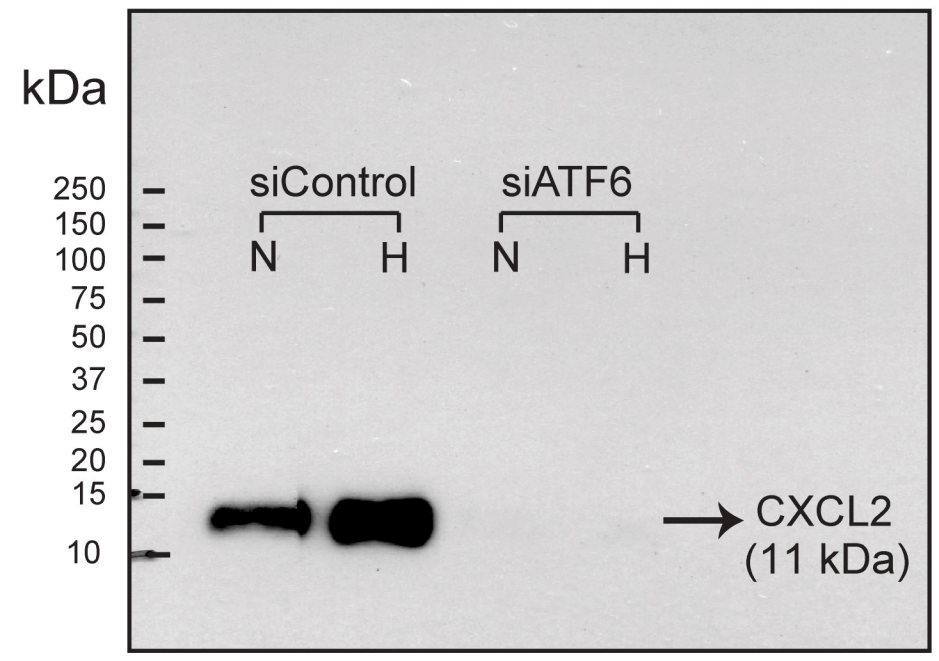

Effect of siATF6 on OIR-induced CXCR2 expression in mice pups

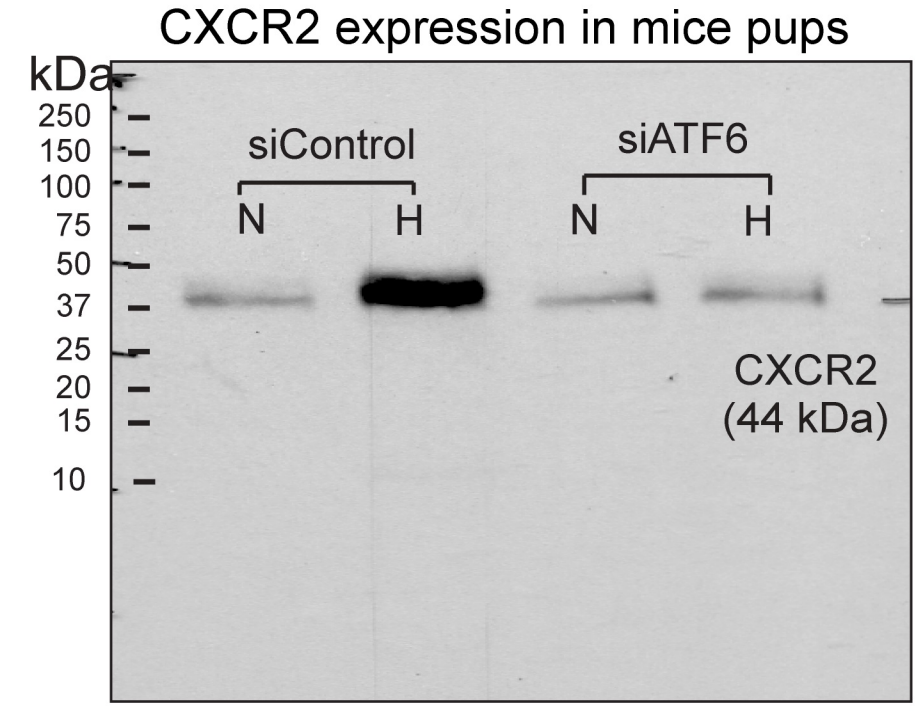

Effect of siATF6 on OIR-induced ATF6 expression in mice pups

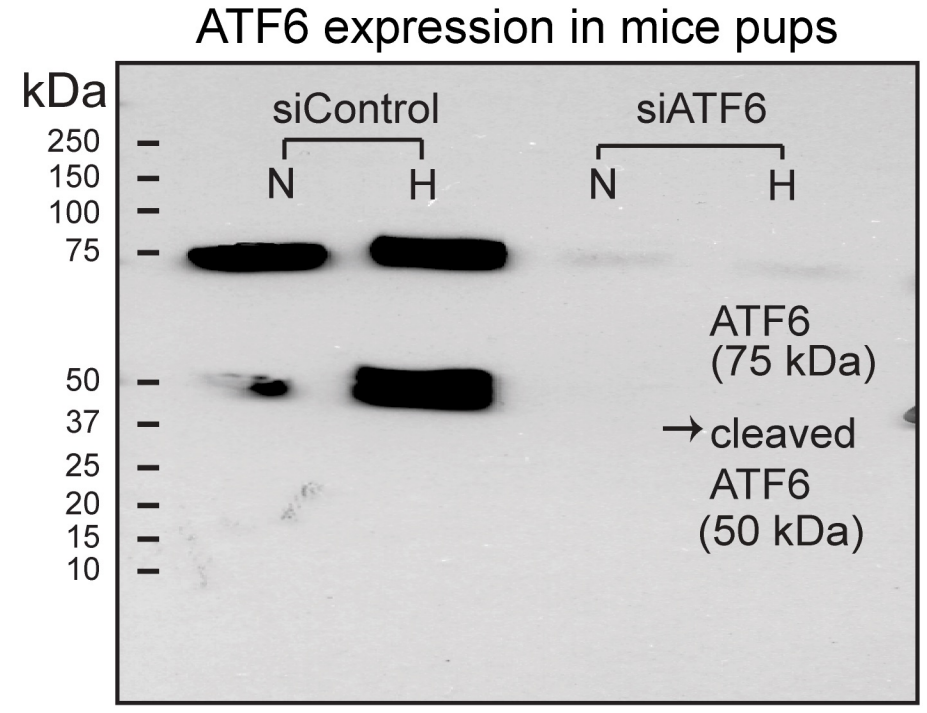

Normalization to β-Actin

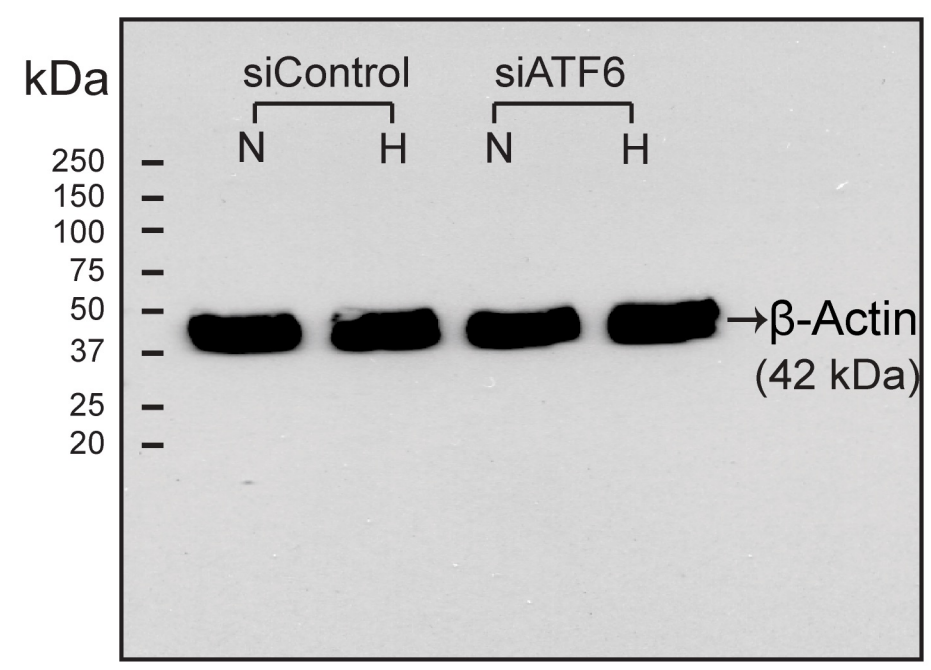

Figure 10A (right panel)

Effect of siGRP78 on OIR-induced CXCL1 expression in mice pups

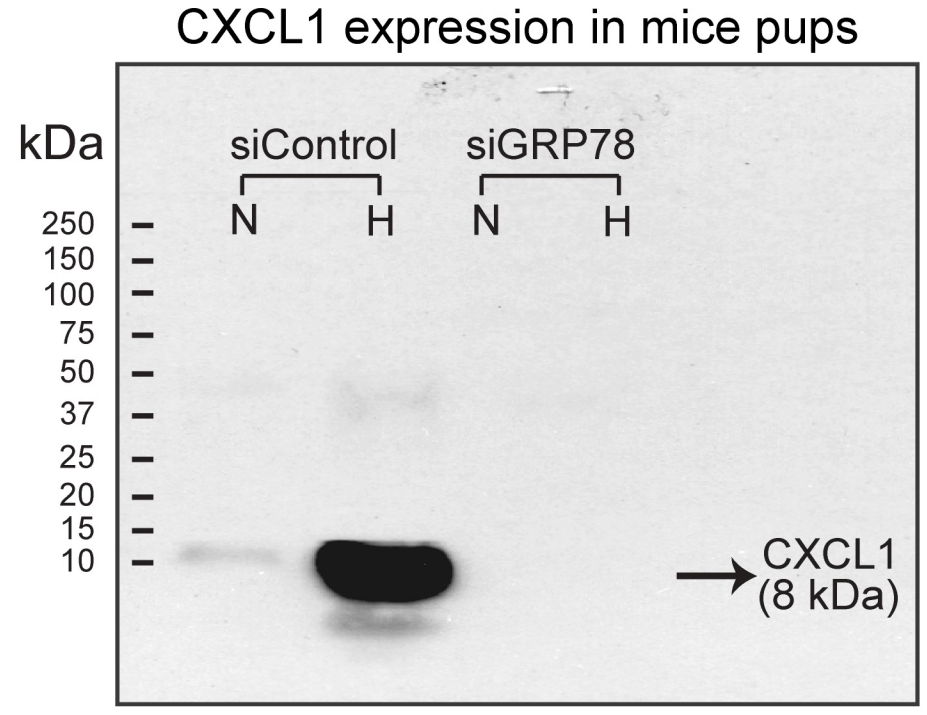

Effect of siGRP78 on OIR-induced CXCL2 expression in mice pups

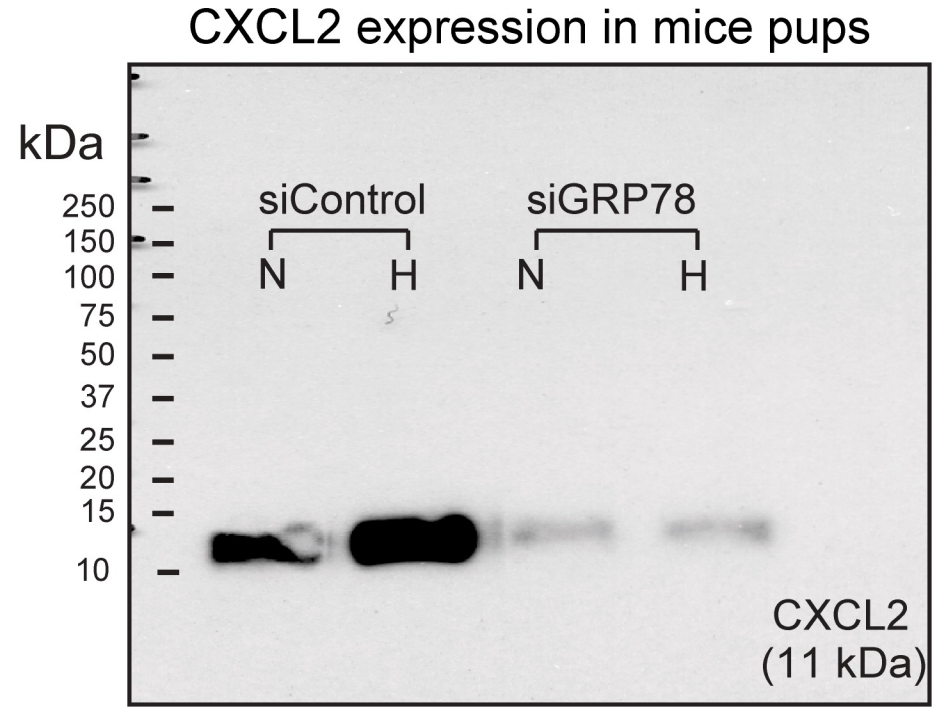

Effect of siGRP78 on OIR-induced CXCR2 expression in mice pups

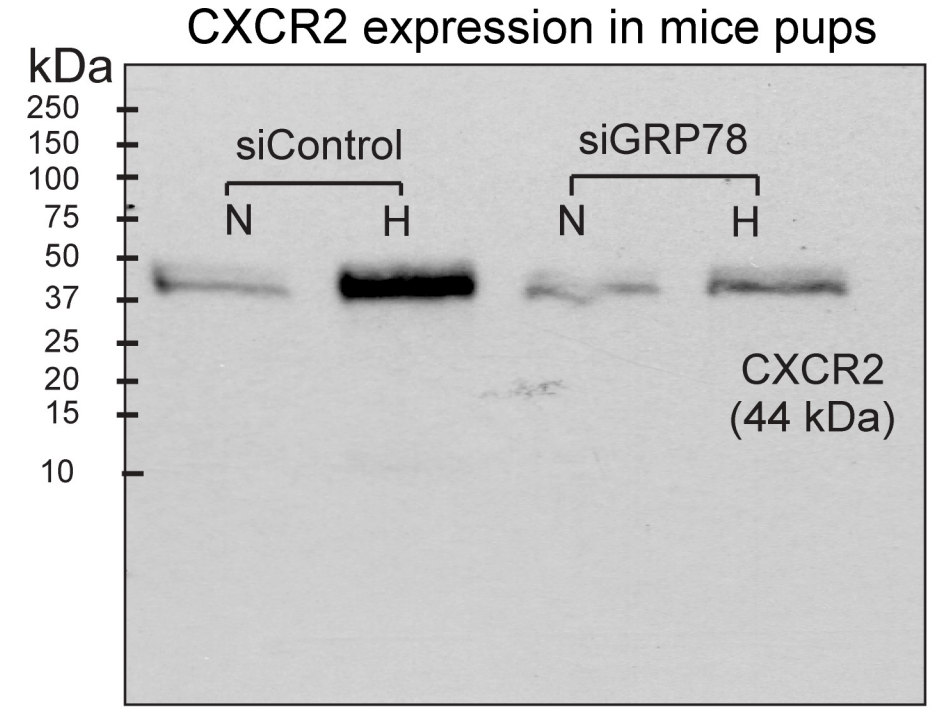

Effect of siGRP78 on OIR-induced GRP78 expression in mice pups

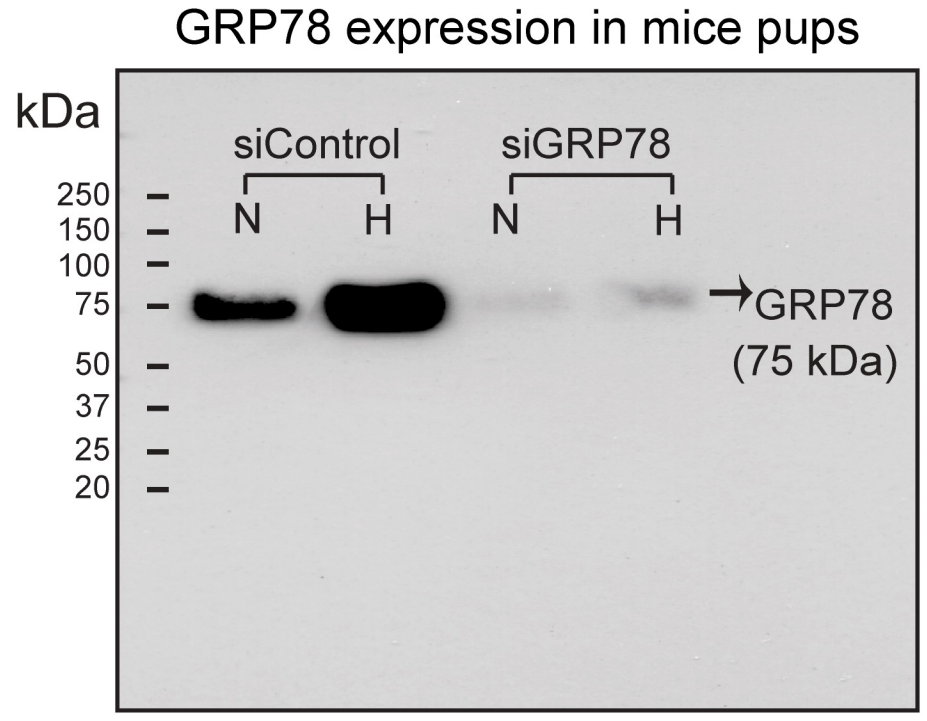

Normalization to β-Actin

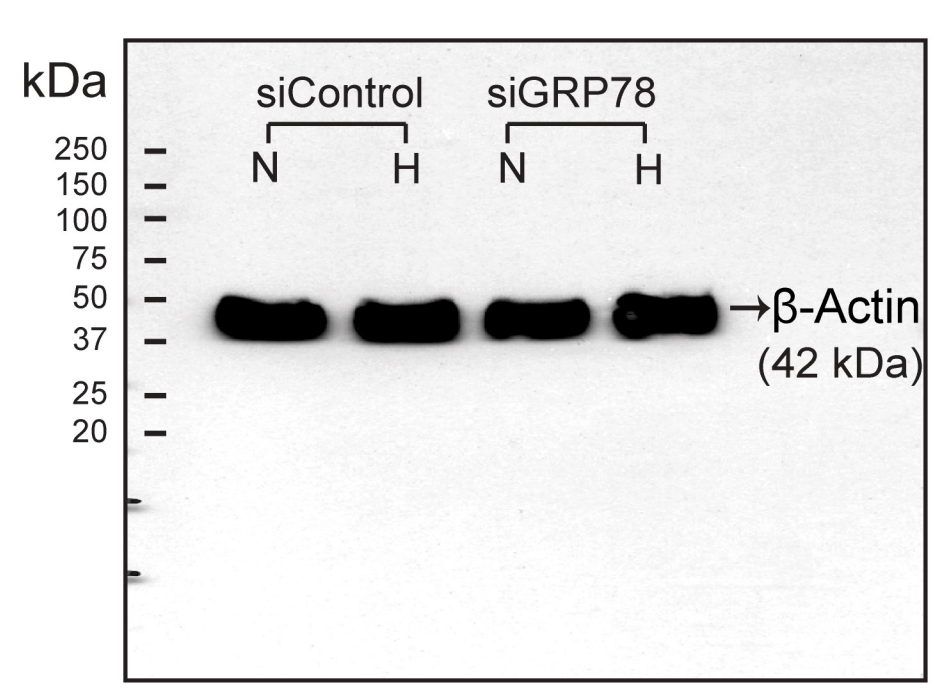

Figure 10B (left panel)

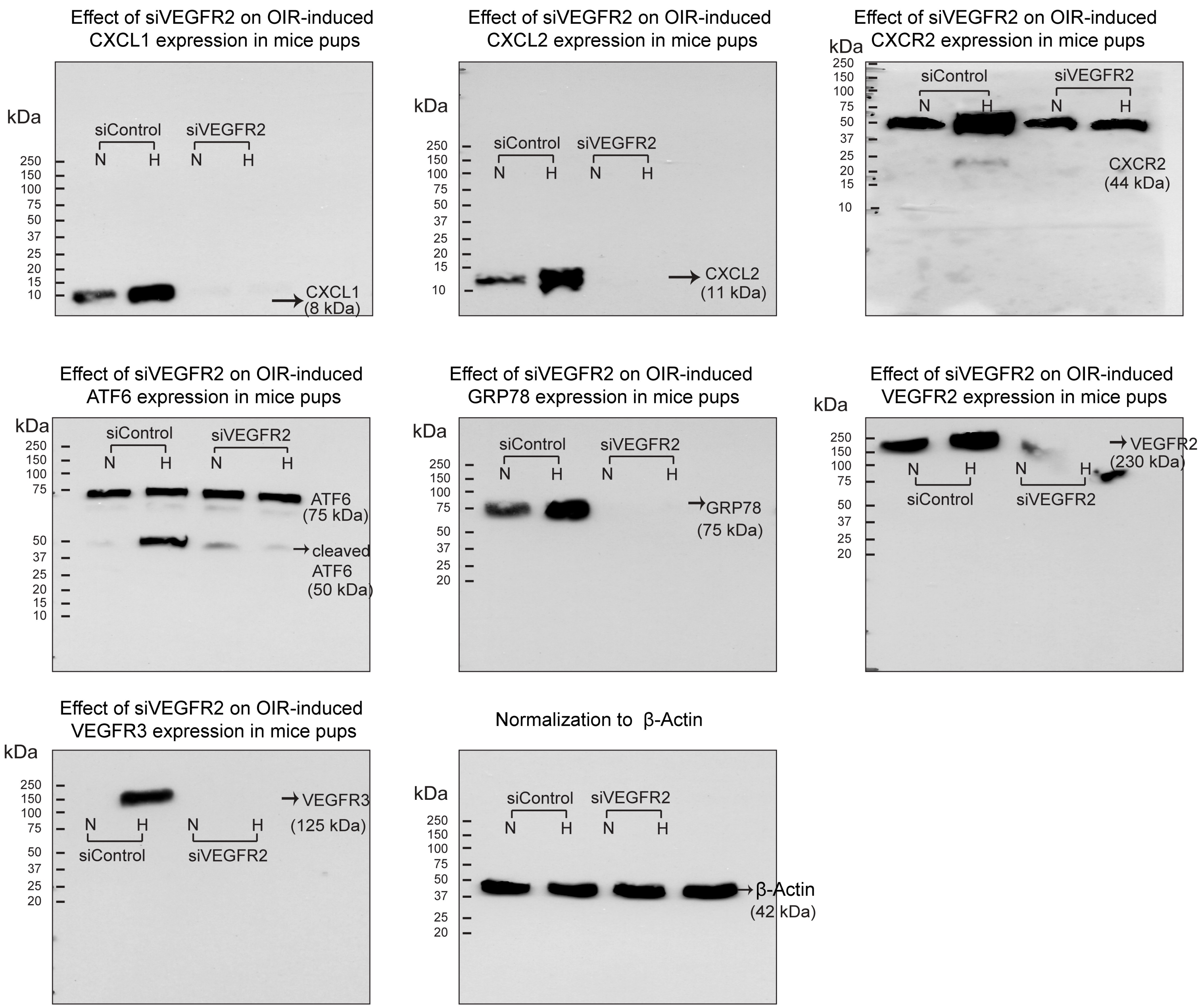

Figure 10B (right panel)

Effect of siVEGFR3 on OIR-induced CXCL1 expression in mice pups

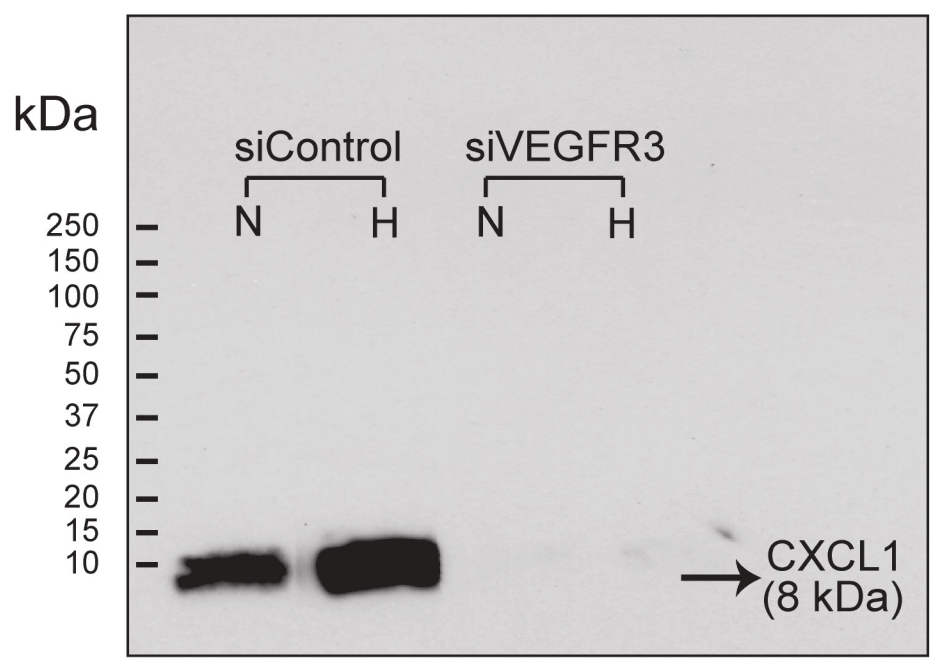

Effect of siVEGFR3 on OIR-induced CXCL2 expression in mice pups

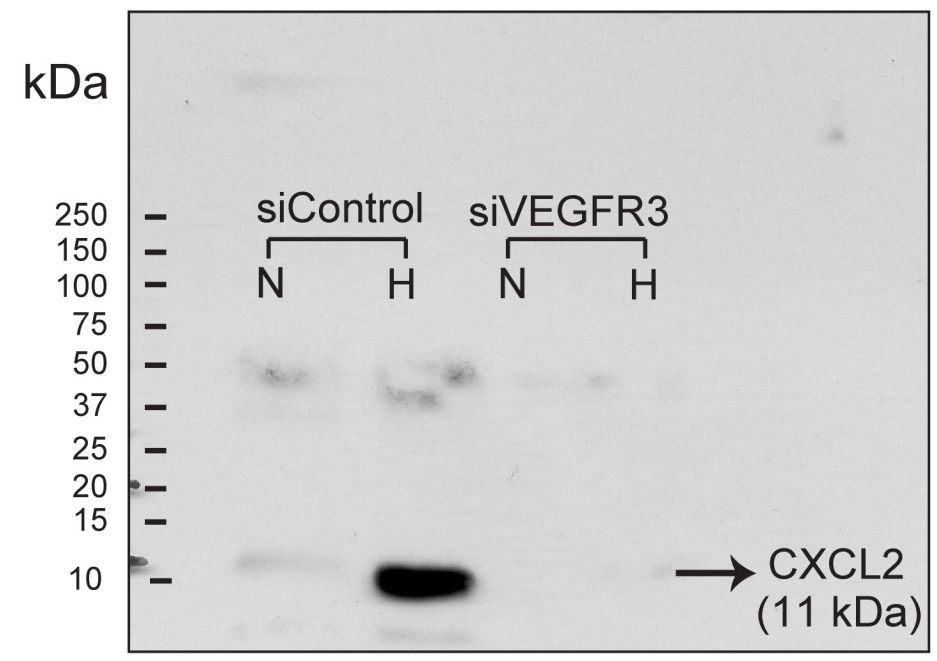

Effect of siVEGFR3 on OIR-induced CXCR2 expression in mice pups

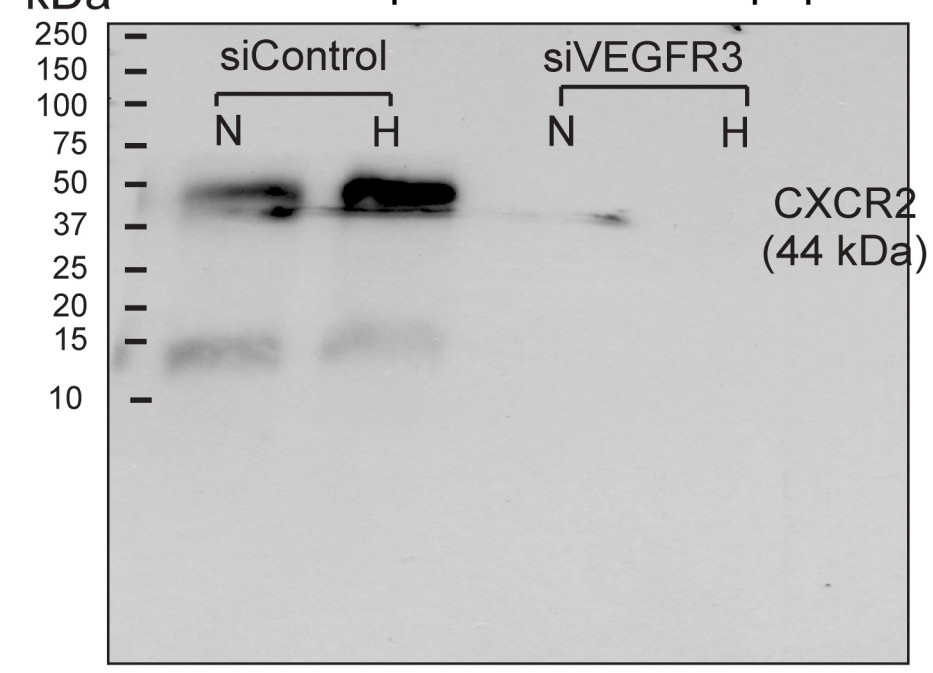

Effect of siVEGFR3 on OIR-induced ATF6 expression in mice pups

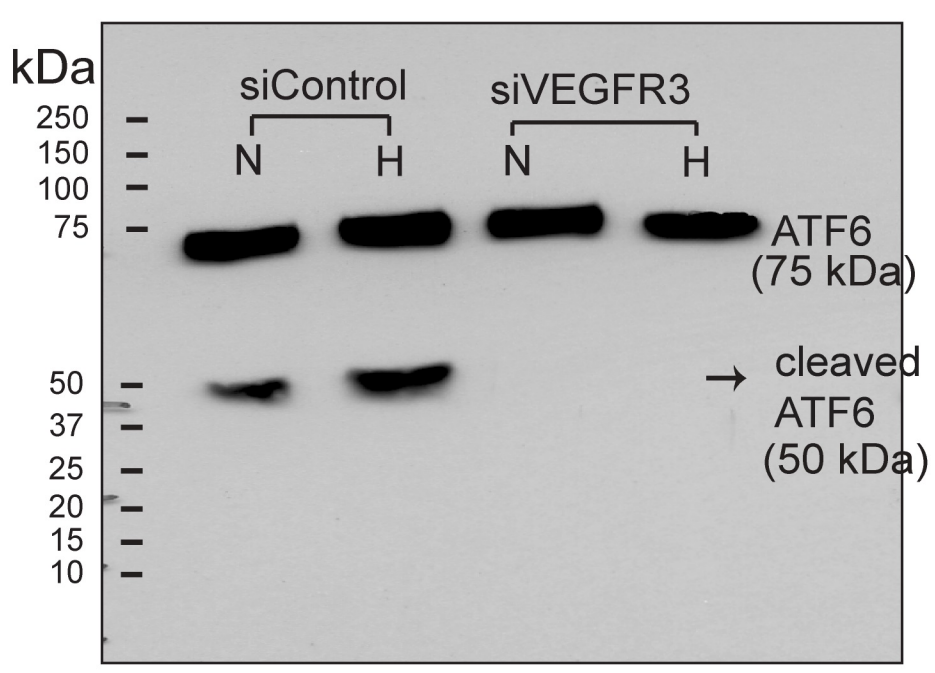

Effect of siVEGFR3 on OIR-induced GRP78 expression in mice pups

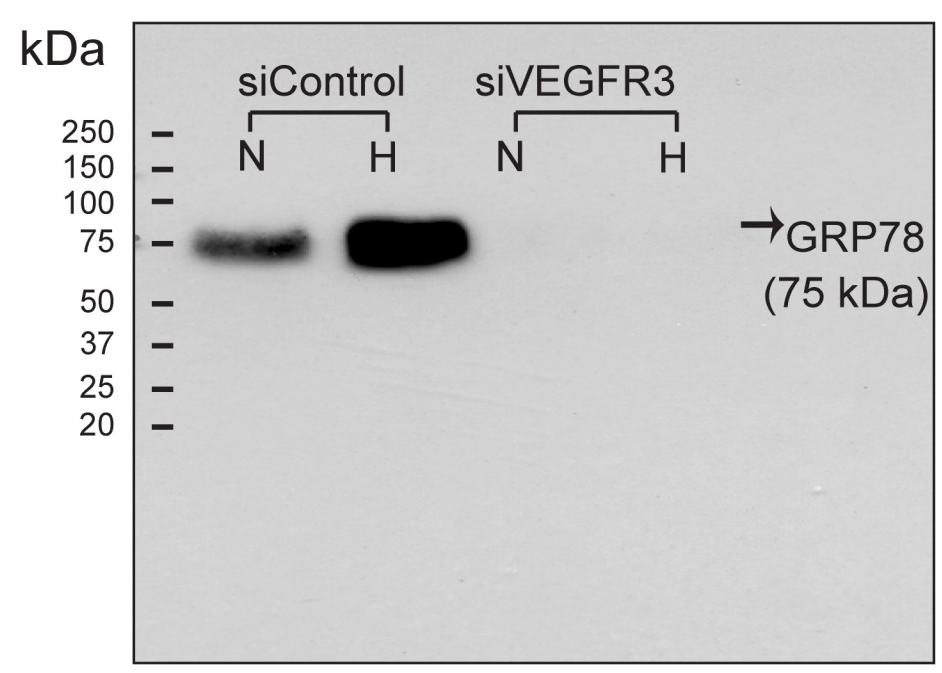

Effect of siVEGFR3 on OIR-induced VEGFR2 expression in mice pups

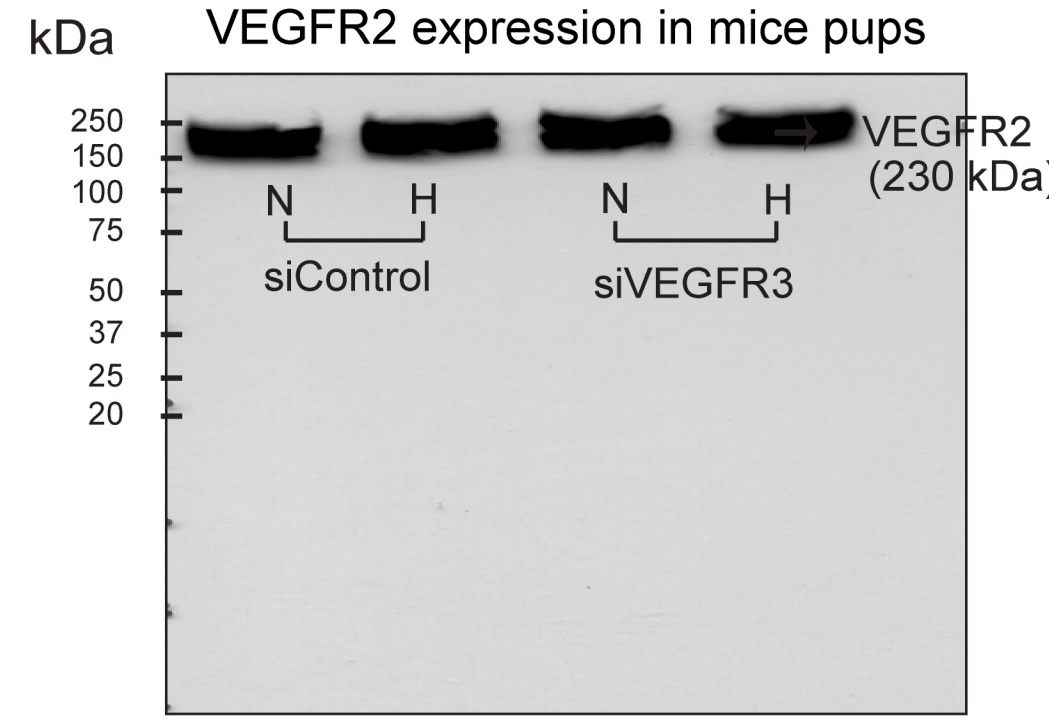

Effect of siVEGFR3 on OIR-induced VEGFR3 expression in mice pups

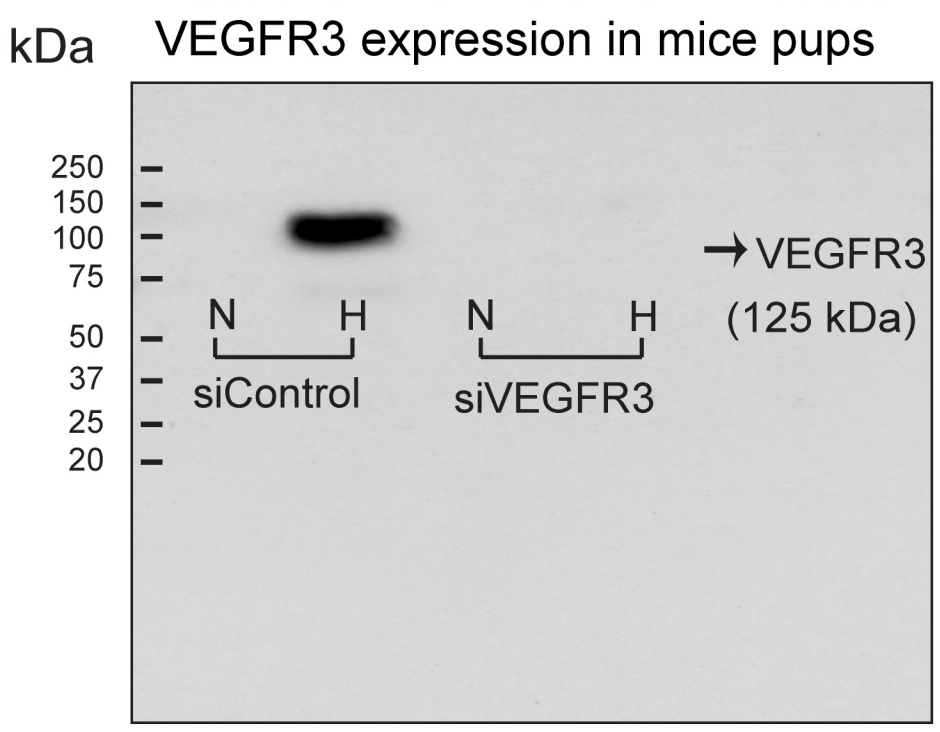

Normalization to β-Actin

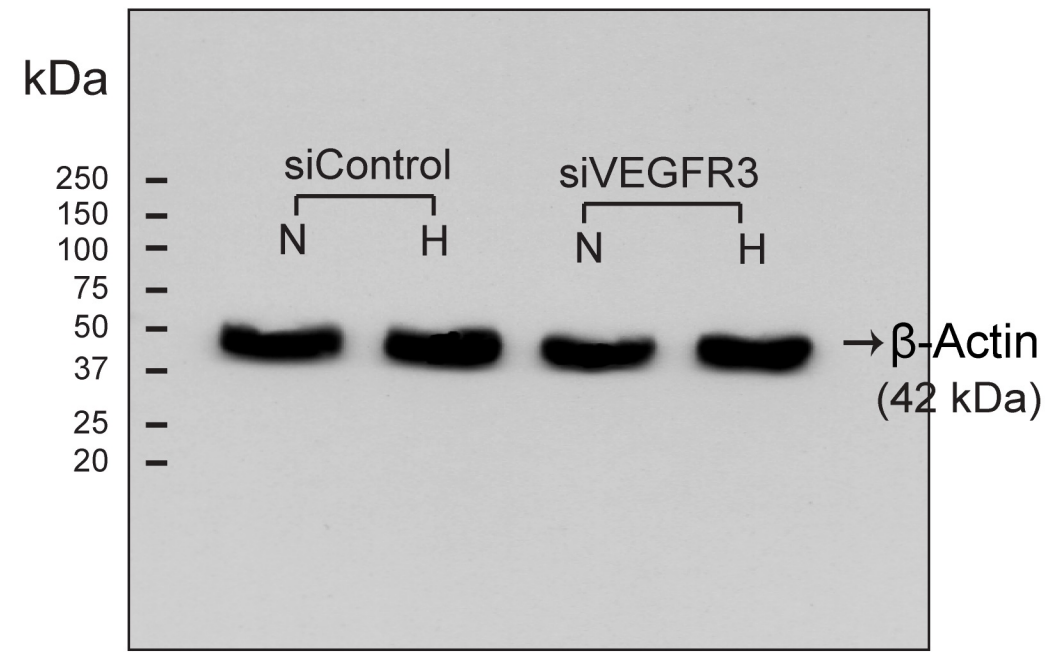

Supplement: Supplementary file 1 [file 10456_2026_10104_MOESM1_ESM.pdf]
